# Untargeted metabolomics analysis of four date palm (*Phoenix* *dactylifera* L.) cultivars using MS and NMR

**DOI:** 10.1007/s13659-023-00406-y

**Published:** 2023-10-23

**Authors:** Shuruq Alsuhaymi, Upendra Singh, Inas Al-Younis, Najeh M. Kharbatia, Ali Haneef, Kousik Chandra, Manel Dhahri, Mohammed A. Assiri, Abdul-Hamid Emwas, Mariusz Jaremko

**Affiliations:** 1 Biological and Environmental Science and Engineering Division, King Abdullah University of Science and Technology (KAUST), Thuwal, 23955-6900 Kingdom of Saudi Arabia; 2 Core Labs, King Abdullah University of Science and Technology (KAUST), Thuwal, 23955-6900 Kingdom of Saudi Arabia; 3 King Abdullah International Medical Research Center (KAIMRC), King Abdullah Int Medical Research Center, NGHA, Jeddah, Kingdom of Saudi Arabia; 4 Biology Department, Faculty of Science, Taibah University, 46423 Yanbu Branch, Yanbu, Saudi Arabia; 5 Department of Pharmacology and Toxicology, College of Pharmacy, King Saud University, Riyadh, Saudi Arabia; 6 Smart-Health Initiative and Red Sea Research Center, Division of Biological and Environmental Sciences and Engineering, King Abdullah University of Science and Technology, P.O. Box 4700, 23955-6900 Thuwal, Saudi Arabia

**Keywords:** Metabolomics, GC–MS, UHPLC-MS, NMR, Date palm, Phytochemicals

## Abstract

Since ancient times, the inhabitants of dry areas have depended on the date palm (*Phoenix* *dactylifera* L.) as a staple food and means of economic security. For example, dates have been a staple diet for the inhabitants of the Arabian Peninsula and Sahara Desert in North Africa for millennia and the local culture is rich in knowledge and experience with the benefits of dates, suggesting that dates contain many substances essential for the human body. Madinah dates are considered one of the most important types of dates in the Arabian Peninsula, with Ajwa being one of the most famous types and grown only in Madinah, Saudi Arabia. Date seeds are traditionally used for animal feed, seed oil production, cosmetics, and as a coffee substitute. Phytochemical compounds that have been detected in date fruits and date seeds include phenolic acids, carotenoids, and flavonoids. Phenolic acids are the most prevalent bioactive constituents that contribute to the antioxidant activity of date fruits. The bioactive properties of these phytochemicals are believed to promote human health by reducing the risk of diseases such as chronic inflammation. Ajwa dates especially are thought to have superior bioactivity properties. To investigate these claims, in this study, we compare the metabolic profiles of Ajwa with different types of dates collected from Saudi Arabia and Tunisia. We show by UHPLC-MS that date seeds contain several classes of flavonoids, phenolic acids, and amino acid derivatives, including citric acid, malic acid, lactic acid, and hydroxyadipic acid. Additionally, GC–MS profiling showed that date seeds are richer in metabolite classes, such as hydrocinnamic acids (caffeic, ferulic and sinapic acids), than flesh samples. Deglet N fruit extract (minimum inhibitory concentration: 27 MIC/μM) and Sukkari fruit extract (IC_50_: 479 ± 0.58μg /mL) have higher levels of antibacterial and antioxidative activity than Ajwa fruits. However, the seed analysis showed that seed extracts have better bioactivity effects than fruit extracts. Specifically, Ajwa extract showed the best MIC and strongest ABTS radical-scavenging activity among examined seed extracts (minimum inhibitory concentration: 20 μM; IC_50_: 54 ± 3.61μg /mL). Our assays are a starting point for more advanced in vitro antibacterial models and investigation into the specific molecules that are responsible for the antioxidative and anti-bacterial activities of dates.

**Graphical Abstract:**

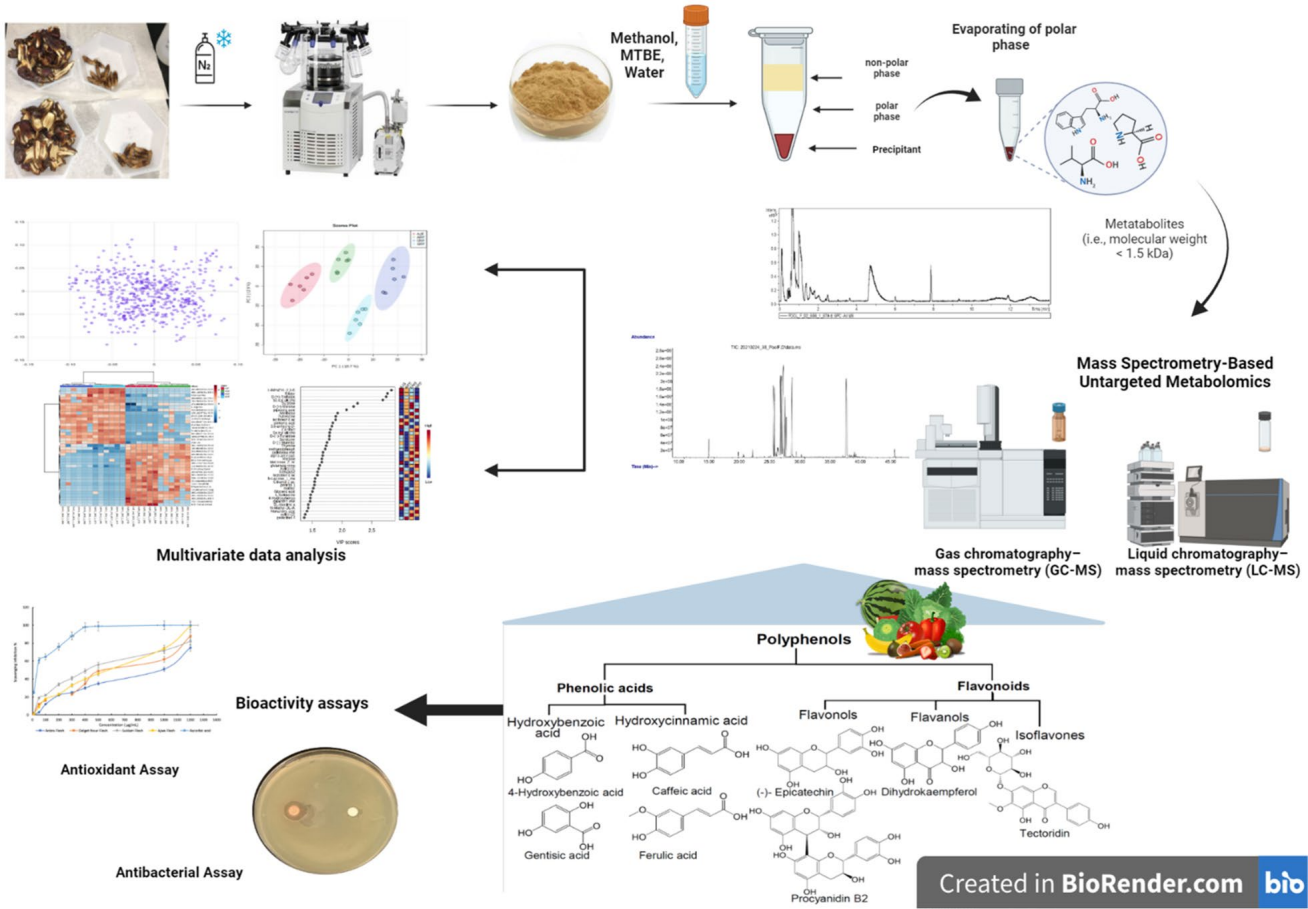

**Supplementary Information:**

The online version contains supplementary material available at 10.1007/s13659-023-00406-y.

## Introduction

As one of the earliest domesticated fruit trees, the date palm (*Phoenix* *dactylifera* L.) is integral to the history and culture of the peoples inhabiting desert regions such as the Arabian Peninsula [1, 2]. For a long time, the date palm has been a staple crop providing food, environmental and economic security in desert regions, thanks to the plant’s hardiness and the numerous beneficial properties of its fruits and seeds, including medicinal, environmental, nutritional, commercial, and economic benefits. Date seeds are traditionally used as animal fodder, for oil production, and in cosmetics, as well as a coffee substitute [3–6]. Date fruits develop through four distinct stages, including an immature stage, also known as kimri, and three stages of maturation, Khalal, Rutab, and Tamar [7]. The nutritional values of date fruits are characterized by low protein and fat content, but the fruits are rich in carbohydrates, primarily monosaccharides (glucose and fructose), dietary fibers, vitamins, and minerals, notably B vitamins and potassium. However, the sugar content varies depending on the date type and fiber content. On the other hand, date seeds are higher in protein and fat content than the date flesh, but like the fruit, also rich in dietary fiber [8].

In addition to these traditionally recognized properties of dates, the date fruit contains high levels of what we today know as phytochemicals, including carotenoids, polyphenols (e.g., phenolic acids, flavonoids, isoflavones, and lignans), tannins, and sterols [3, 7, 9–11], all of which form part of the plant’s immune system and are thought to be beneficial also to the human (and animal) immune system. It is also reported that date seeds contain a wide range of phytochemicals (e.g., flavonoids and alkaloids, anthraquinone, saponin, terpenoids and tannin) [12]. In both animal and clinical human studies, the phytochemicals derived from date fruits show various bioactive properties, such as free radical scavenging, antioxidation, antimicrobial activity, antimutagenesis, anti-inflammation, antihyperlipidemia effects, hepatoprotection, gastroprotection, and anticancer activity [11, 13–18]. However, many factors affect the nutritional and phytochemical properties of date palm fruits. These factors include environmental conditions such as irrigation and water quality, drought, insect pests, pesticides, fertilizers, and geographical location, as well as physical and physiological characteristics such as maturity stage and type [10, 19–22].

A well-balanced diet is essential for preventing metabolic disorders like obesity and diabetes, which are often attributed to oxidative stress, a condition in which prooxidants predominate over antioxidants [23–25]. Antioxidants, especially polyphenols derived from natural products, have demonstrated potential in the treatment and prevention of diseases such as diabetes retinopathy, cardiovascular disease, and various cancers, and it is assumed that adding foodstuffs, such as dates which are high in antioxidants and other beneficial nutrients to the diet help support the body’s own defenses against disease [26–29].

While the date palm has long been recognized for its health benefits, it is only recently that the technology of modern science has made it possible to gain insight into these health-promoting properties at the molecular level. The molecular constituents of dates and seeds mentioned above, such as phytochemicals, are thought to impact diverse human metabolic pathways, resulting in specific health benefits to the human physiology [30]. For instance, the abovementioned compounds have been linked to improvement ins anti-inflammatory processes and regulation of insulin production [11, 31].

Understanding the human metabolic pathways significant for individual health, and their interactions for example with date palm components, has been greatly enhanced by metabolomics research as metabolomics provide useful information for both plant research and human clinical trials, for example enabling the identification and analysis of bioactive compounds in plants and providing insight into their internal mechanisms of action. At the same time, metabolomics offers complementary, detailed and precise methods for investigations of the needs and responses at the molecular level of patients in the context of clinical trials, offering crucial insights into potential and applied methods for the management, prevention, and treatment of disease [32]. Connecting the fields of plant science and human health in a multidisciplinary approach has great potential, therefore, in teaching us how to best make use of for example date palm fruits (and seeds) and their metabolites to improve overall well-being [33–37].

Metabolomics commonly utilizes different analytical platforms including nuclear magnetic resonance (NMR) [38–40], Fourier-transformed infrared (FT-IR) spectroscopy [41, 42], and mass spectrometry (MS) [43]. MS is usually combined with other analytical tools such as gas chromatography (GC–MS) or liquid chromatography (LC–MS) [42, 44].

Using these tools, the objective of the study we present here is to comprehensively examine the nutritional and phytochemical properties of date palm fruits and seeds by conducting a detailed investigation of four popular date types, all under the same conditions. The date types examined, are Ajwa (AJ), Anbra (AR), Sukkari (SR), and Deglet Nour (DN) dates. We used the MS&NMR untargeted metabolomics approach in this study to elucidate the metabolic profiles and uncover the complicated chemical composition of these date varieties. Furthermore, we evaluated the antioxidant and antibacterial properties of date extracts using different bioactivity assays, such as the ABTS test, disc diffusion assay, and MIC assay. In addition, our aim with this study is to contribute to a deeper understanding of how these chemicals may influence overall well-being, particularly in terms of their antioxidant and antibacterial characteristics. The outcomes of this comprehensive study will hopefully provide important insights into the nutritional and health-related properties of date palm fruits and seeds and provide a foundation for further research into their known and potential other benefits to human nutrition and health.

## Results

### UHPLC-MS profiling of polar to semi-polar secondary metabolites in P. dactylifera seed and flesh extracts

#### Metabolic profiling

**Fig. 1:**
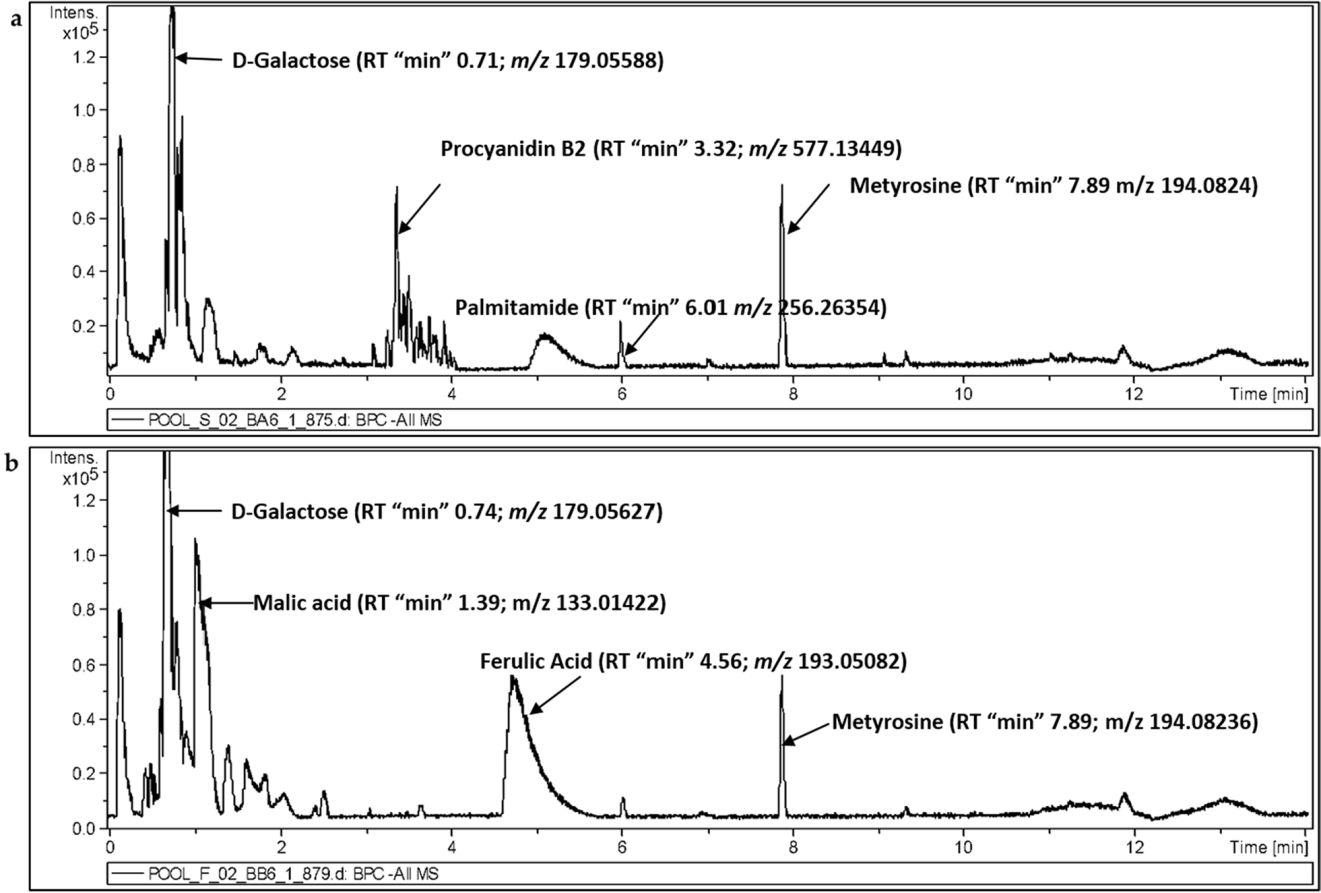
Base peak intensity UPLC-MS chromatograms of pooled samples under negative ionization mode. **a** A chromatogram of date seeds. **b** A chromatogram of pooled date flesh

UHPLC-ESI-Q-TOF–MS was used to analyze the secondary metabolites in date flesh and seed extracts (Fig. 1). Various classes of metabolites were separately analyzed under ESI positive and negative ion modes. A total of 96 known metabolites were identified in the flesh extracts analysis of four date palm types. The annotated metabolites in the seed samples totaled 98 compounds. These identified chemical compounds include organic acids, flavonoids, phenolic acids, amino acids, amines, fatty acids, and sphingolipids. All know detected metabolites are listed in Additional file 1: Tables S1 and S2.

#### Identification of polyphenols

The UHPLC-MS revealed that date seeds and flesh contain several sub-classes of phenolic acids and flavonoids (or derivatives). For instance, hydroxybenzoic acid, a subclass of phenolic acids was detected in seed and flesh profiling, greatly in the seed analysis; 4-hydroxybenzoic acid was detected in date seeds (*m/z* 139.03906) and flesh; in addition, gentisic acid (*m/z* 153.01932) and vanillic acid (*m/z* 169.0497) were detected in date seeds.

The dates profiling (all 4 types) revealed presence of hydroxycinnamic acids and their derivatives, another subclass of phenolic acids, as well as caffeic acid, which was found in both flesh and seed but appeared at higher levels in the seed analysis. Furthermore, 5-O-caffeoylshikimic acid was detected both in the flesh and seed analyses, while ferulic acid was found only in the flesh profiling.

Flavonoids such as (-)-epicatechin (C1_5_H_14_O_6_), was detected in high abundance in all date seeds, but especially in Sukkari seed. However, epicatechin was not detected in date flesh. Other flavanols such as procyanidin B2 (C_30_H_26_O_12_), were also relatively abundant in date seeds, especially in the Sukkari variety. Additionally, dihydrokaempferol (*m/z* 287.05597; predicted formula C_15_H_12_O_6_) and tectoridin (*m/z* 461.10875; with predicted formula C_22_H_22_O_11_), were only detected in date seeds. Table 1 summarizes the polyphenol compounds that were detected via UHPLC-MS in the four varieties of date seeds and flesh.

**Table 1:** Summary of identified polyphenol metabolites detected by UHPLC-MS in four varieties of date seeds and flesh

Identified polyphenols		Predicted chemical formula	UPLC-ESI–MS
*1—Phenolic* *acids* A Hydroxybenzoic acids	4-hydroxybenzoic acid	C_7_H_6_O_3_	Flesh & Seed
	Gentisic acid	C_7_H_6_O_4_	Seed
	Vanillic acid	C_8_H_8_O_4_	Seed
B Hydroxycinnamic acids and derivatives	Caffeic acid	C_9_H_8_O_4_	Seed & Flesh
	Ferulic acid	C_10_H_10_O_4_	Flesh
	5-O-Caffeoylshikimic acid	C_16_H_16_O_8_	Seed & Flesh
*2—Flavonoids* A Flavanols	Epicatechin	C_15_H_14_O_6_	Seed
	Procyanidin B2	C_30_H_26_O_12_	Seed
B Flavonols	Dihydrokaempferol	C_15_H_12_O_6_	Seed
C Isoflavonoids	Tectoridin	C_22_H_22_O_11_	Seed

#### Multivariate statistical analysis of UHPLC–MS data

To identify the differences and similarities in the chemical compositions of the four tested date cultivars, UHPLC–MS spectra datasets were processed using MetaboAnalyst to optimize peak alignment, detection, and quantification. The resulting peak intensity datasets of the merged positive and negative ion modes were sum normalized, log transformed, and auto scaled for a basic visual examination and data quality check. The significant loadings were cross-confirmed using the Kruskal–Wallis ANOVA and an adjusted P-value (FDR) cutoff of 0.05 (Additional file 1: Fig. S1).

**Fig. 2:**
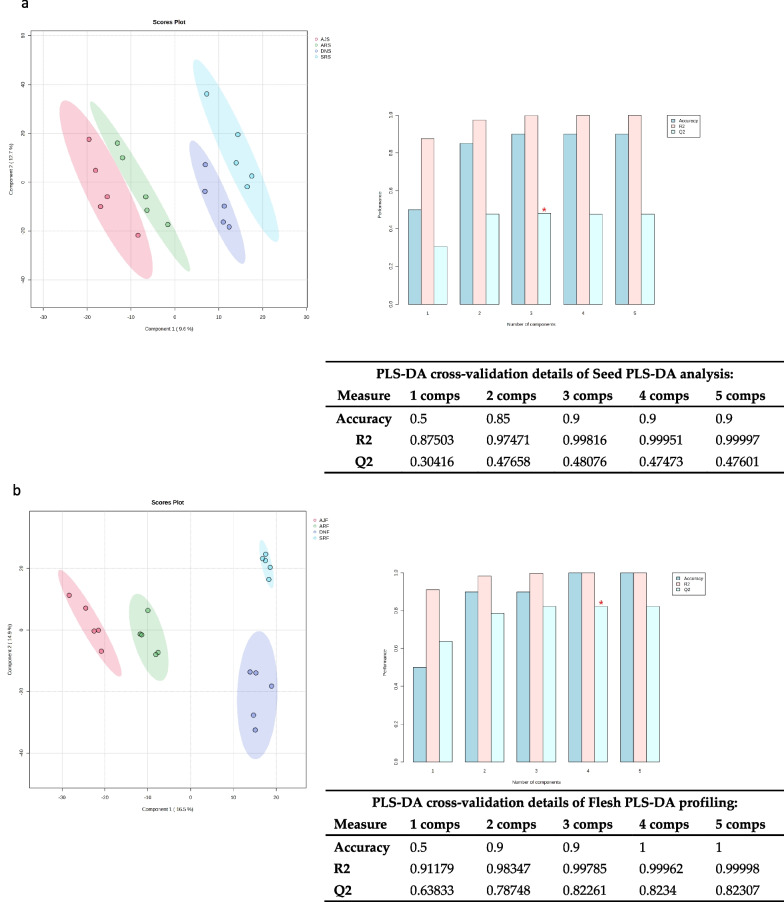
PLS-DA of UHPLC-MS data from four *P. dactylifera* varieties (n = 5). The score plots show the metabolome clusters of date samples, plotting the first and second components with the variance percentages of the data explained by components, in two-dimensional space. **a** Score plot displaying the clustering of metabolites in seed samples. Component 1 (9.6% variance) and Component 2 (12.7% variance) reveal distinct patterns among different date varieties of seed groups. **b** Score plot displays metabolome clusters in flesh samples. Component 1 (16.5% variance) and Component 2 (14.9% variance) illustrate discernible variations in metabolite profiles of fruit flesh across date varieties. Furthermore, the assessment of the PLS-DA model is supported by R^2^ and Q^2^ values both exceeding 0.7, confirming the validity and reliability of the results obtained through PLS-DA analysis

**Fig. 3.**
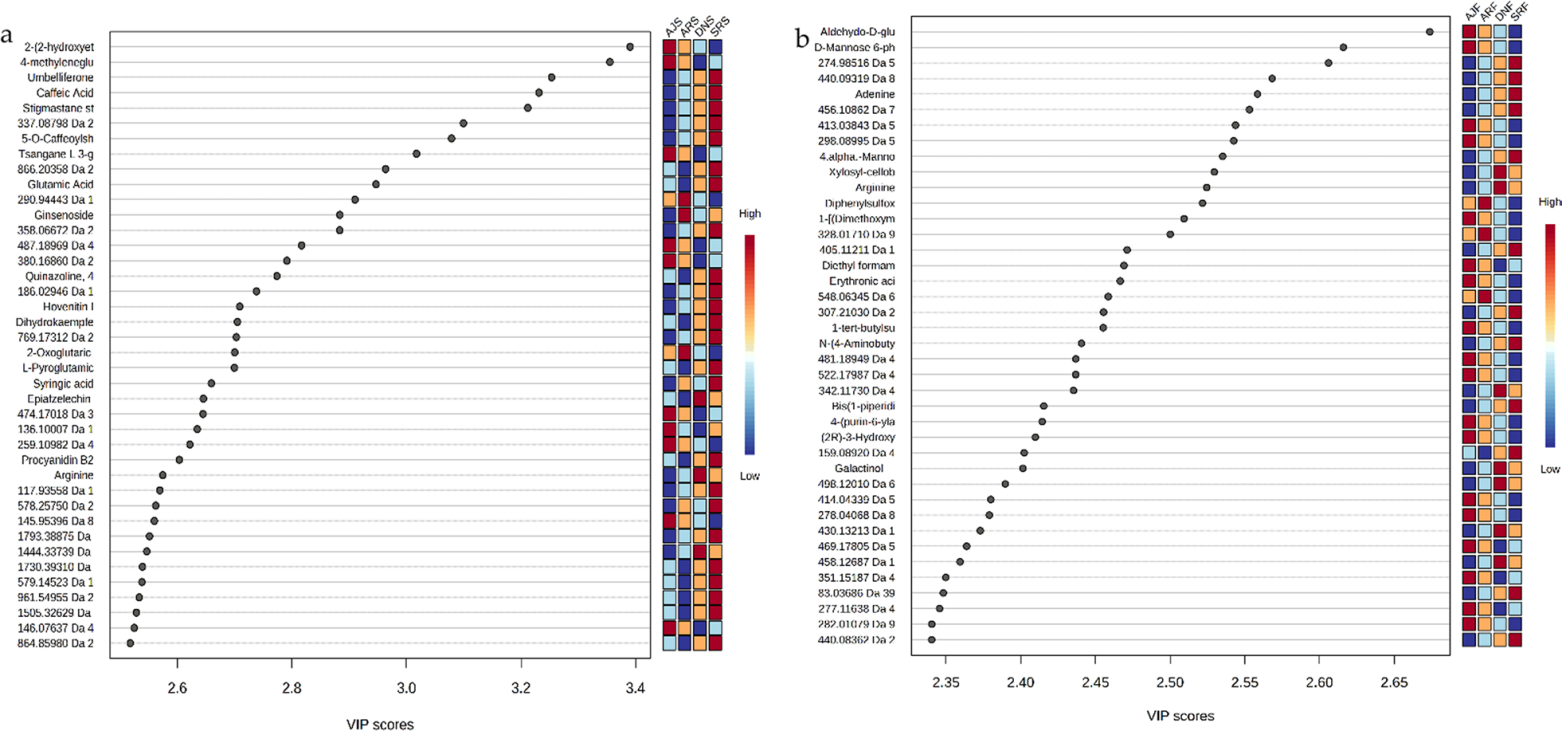
40 VIP scores in **a** pooled seed samples and **b** in pooled flesh samples. The x-axis displays the correlation of the score, and the y-axis corresponds to the UHPLC-MS peak number from the peak index. Color bars exhibit the median intensity of variables in the respective groups

Figure 2 shows the PLS-DA of five biological replicates from the same date palm type, in which the date varieties (AJ, AR, SR, and DN) cluster together. In general, the PLS-DA score plots of the chemical compositions for seed and flesh show considerable clustering and separation within dates varieties (Fig. 2a, b) as well as PLS-DA cross-validation data shown in (Figs. 2c, d). The top 40 metabolite features (variables) for each type of date were calculated based on variable importance in projection (VIP) scores from the PLS-DA (Fig. 3). Figure 3a shows an overview of 40 VIP variable compounds in the seed samples, which included some annotated compounds at high levels in SRS, such as umbelliferone (C_9_H_6_O_3_) at *m/z* 163.0392, caffeic acid (C_9_H_8_O_4_) at *m/z* 181.0499, and procyanidin B2 (C_30_H_26_O_12_) at *m/z* 577.13405. In addition, tsangane L 3-glucoside (C_19_H_34_O_7_) was found to be present in relatively high amounts in AJS compared to other seed samples. The VIP score of flesh samples (Fig. 3b) confirms that most components detected in the flesh parts are carbohydrates. For example, aldehydo-d-glucose (C_6_H_12_O_6_) was relatively high in AJF, and xylosyl-cellobiose (C_16_H_28_O_16_) was higher in DNF, compared to other flesh samples.

**Fig. 4:**
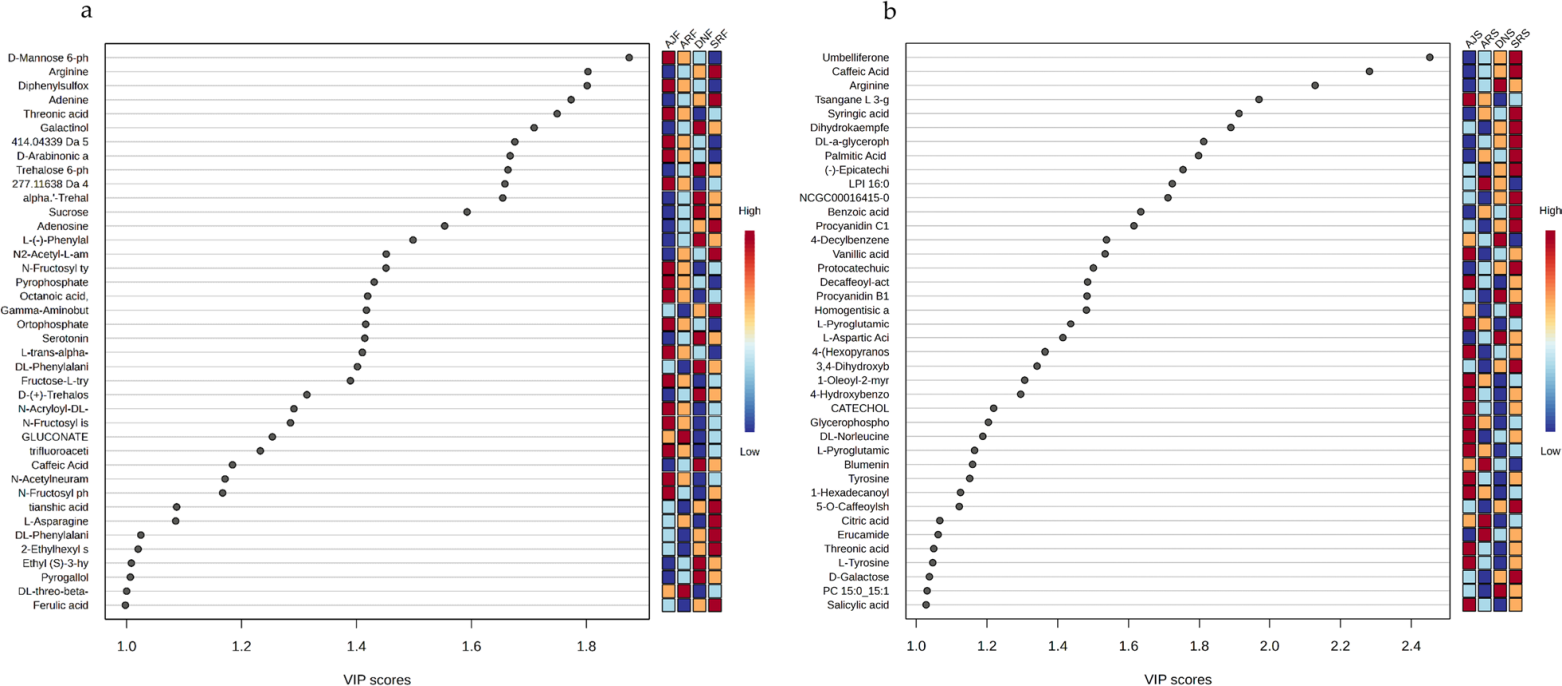
VIP analysis identifies 40 annotated compounds: **a** VIP analysis highlighting 40 annotated compounds of interest with significant relevance for flesh profiling and **b** the same for seed analysis

**Fig. 5:**
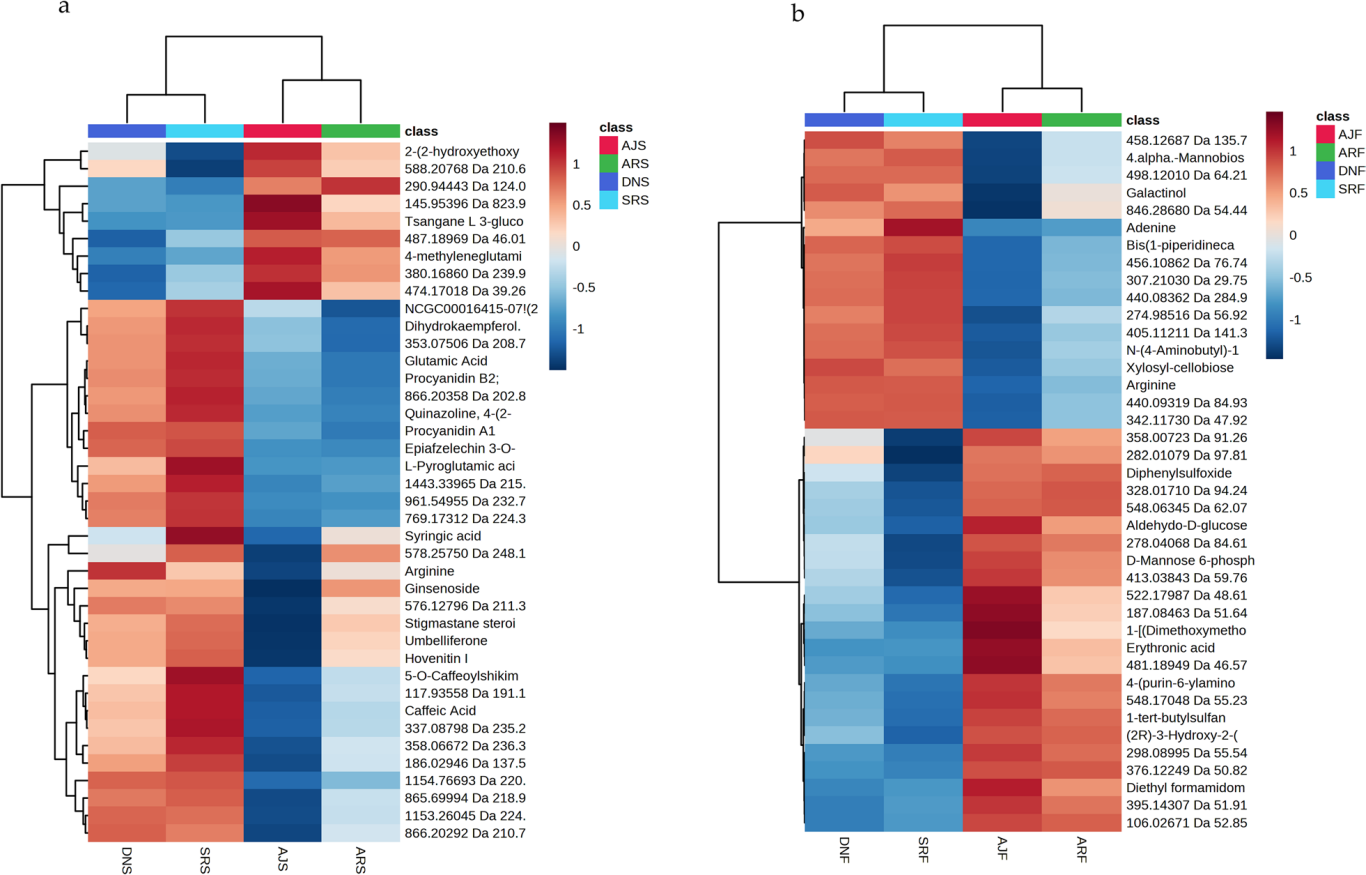
Hierarchical cluster analysis (HCA) of the four date cultivars based on UHPLC-MS data using MetaboAnalyst software for **a** seed samples and **b** flesh samples. The heatmap shows average of date varieties group in rows and metabolite features in columns. Each colored cell in the heatmap corresponds to the concentration of the indicated metabolite. Red indicates high concentration and blue indicates low concentration

**Fig. 6:**
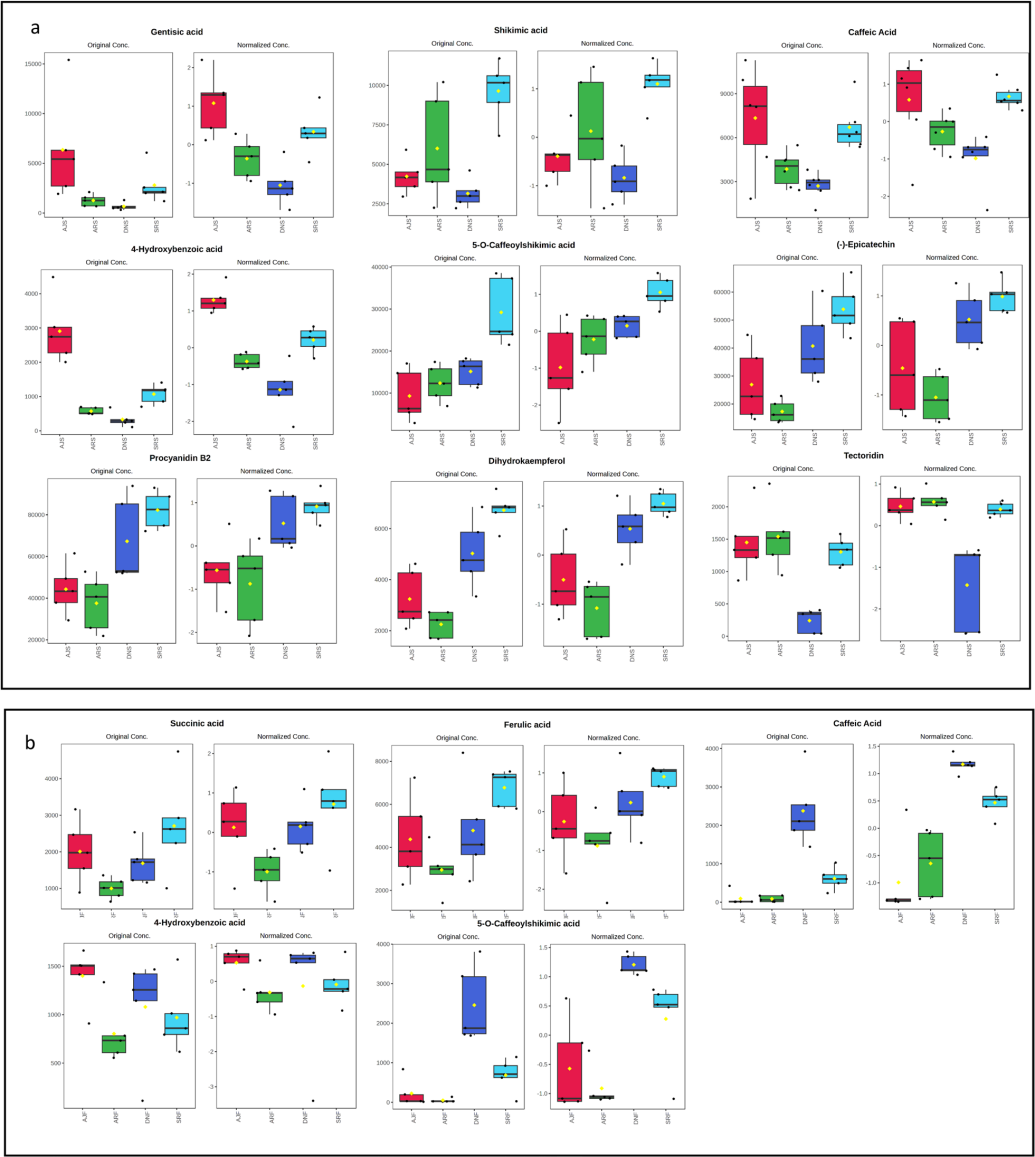
Post-hoc analysis reveals different metabolite abundance among date palm groups, where **a** is for the seed groups and **b** is for the flesh groups. This figure illustrates the results of post-hoc tests following an ANOVA analysis, highlighting the relative abundance of significant metabolites or features within each group. The associated p-values for these metabolites can be found in tables S5-S8, providing insight into the statistical significance of these observed differences

In addition, for the annotated compounds in Fig. 4, the VIP analysis identified 40 annotated compounds of interest with significant relevance for (a) seed profiling and (b) for flesh analysis. The heatmap visualization of the 40 VIP-annotated compounds from the flesh profiling analysis are shown in (Additional file 1: Fig. S3a) and that for the seed analysis is shown in (Additional file 1: Fig. S3b). Moreover, clustering heatmaps were employed as an additional tool to assess the variation among date varieties. Based on the similarity of the metabolic patterns as well as the groups of metabolites that drive clustering of the date samples, two groups of dates clustered together, as shown in Fig. 5. The first group contained the Anbra and Ajwa cultivars, and the second group consisted of Sukkari and Deglet N. It is important to mention that the Ajwa and Anbra samples have the same origin (Medina, Saudi Arabia), but Sukkari and Deglet N are from different regions (Hail and Qassim, Saudi Arabia, and Tozeur, Tunisia, respectively). A heatmap analysis of seed samples (Fig. 5a) showed a relatively high abundance of hovenitin I (C_16_H_14_O_8_) and dihydrokaempferol (C_15_H_12_O_6_), especially in SRS. However, there was also an overlap between SRS and DNS clustering (Fig. 5a). Figure 5b shows differentiates metabolites that were highly abundant in the flesh extracts of Sukkari and Deglet N dates compared to Ajwa and Anbra dates, such as galactinol (C_12_H_22_O_11_) and arginine (C_6_H_14_N_4_O_2_). Besides the known component erythronic acid (C_4_H_8_O_5_), there are some unknown metabolites, like *m/z* 276.10911 and *m/z* 415.05067, with predicted chemical formulas of C_11_H_19_NO_7_ and C_16_H_14_O_13_, respectively, which could be significant molecules differentiating AJF from the other varieties (Fig. 5b). Moreover, Fig. 6 shows a post-hoc analysis, which revealed different metabolite abundance among the date palm varieties, where Fig. 6a show data for the seed groups and Fig. 6b show data the flesh groups. These figures illustrate the results of post-hoc tests following an ANOVA analysis, highlighting the relative abundance of significant metabolites or features within each group. The associated p-values for these metabolites can be found in Additional file 1: Tables S5–S8, providing insight into the statistical significance of these observed differences.

### *Metabolite profiling of P. dactylifera seeds and flesh* via *GC–MS*

#### Metabolic profiling

**Fig. 7:**
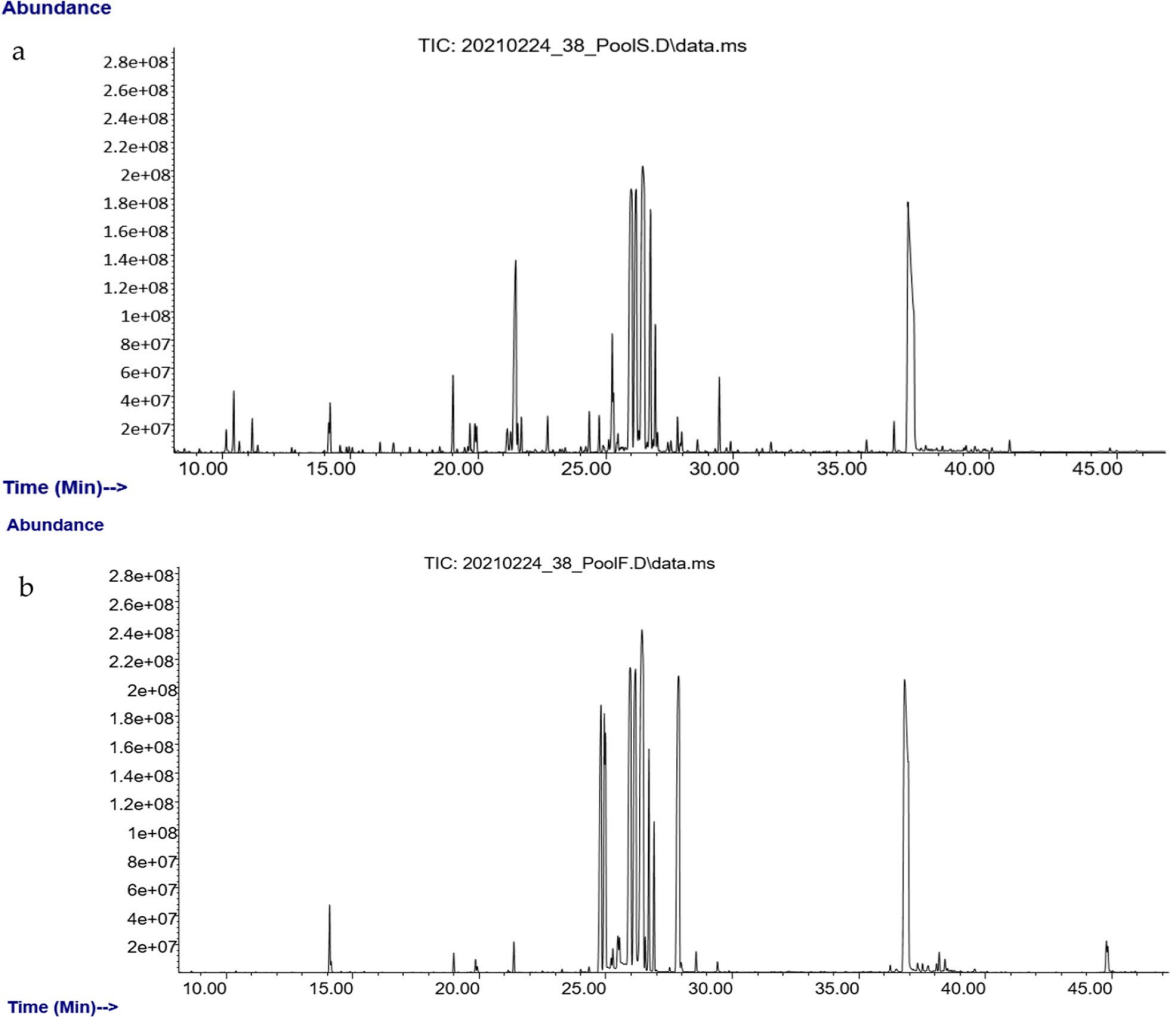
Total-ion GC–MS chromatogram of the pooled sample containing all *P.* *dactylifera* varieties. **a** Seed metabolites. **b** Flesh metabolites

GC–MS was used to profile primary metabolites among the four date types (Fig. 7). As shown in Additional file 1: Tables S1 and S2, the majority of metabolites identified by GC–MS are simple carbohydrates like monosaccharides and oligosaccharides (e.g., there is a high abundance of glucose and fructose in flesh samples among all cultivars). There were fewer chemical constituents in flesh samples than seed samples (e.g., fatty acids; compare Additional file 1: Tables S1 and S2).

Palmitic acid (C_16_H_32_O_2_ with RT min 29.26) and stearic acid (C_18_H_36_O_2_ with RT min 32.23) were found at higher abundance in seeds extracts than flesh extracts, especially in the Ajwa variety. Ajwa seed was also found to have high levels of oleic acid, lauric acid, and citramalic acid. Several other organic acids were also identified via GC–MS, such as oxalic acid and malic acid, which were the predominant organic acids identified in seeds (Additional file 1: Table S1), with Ajwa showing the highest abundance. Similarly, in the flesh analysis, oxalic acid and malic acid were found at high levels specifically in Deglet N, followed by citric acid.

Furthermore, flavonoid compounds, such as catechins (C_15_H_14_O_6,_) were detected at relatively high levels in seeds but not detected in flesh. The results also revealed the presence of dopamine as well as serotonin and gamma aminobutyric acid (GABA), but only in the flesh (Additional file 1: Tables S1 and S2). Table 2 shows a summary of the detected compounds in both seeds and flesh.

**Table 2:** Date palm seed and flesh primary metabolites analyzed by GC–MS (including carbohydrates, amino acids, fatty acids, and polyphenols)

Classification	Seed	Flesh
*Sugars*		
Monosaccharides	** + **	** + **
Oligosaccharides	** + **	** + **
*Amino* *acid* *derivatives*		
Gamma amino acids and derivatives (GABA)	** + **	** + **
Proline and derivatives	** + **	
Glutamic acid and derivatives	** + **	** + **
*Fatty* *acid*		
Long-chain fatty acids	** + **	** + **
Hydroxy fatty acids	** + **	
Lineolic acids and derivatives	** + **	
*Polyphenols*		
Hydroxycinnamic acids	** + **	** + **
Catechins	** + **	
Hydroxybenzoic acids	** + **	** + **
Shikimic acids and derivatives	** + **	** + **
*Dopamine*		** + **
*Serotonin*	** + **	** + **

#### Statistical analysis of GC–MS data

**Fig. 8:**
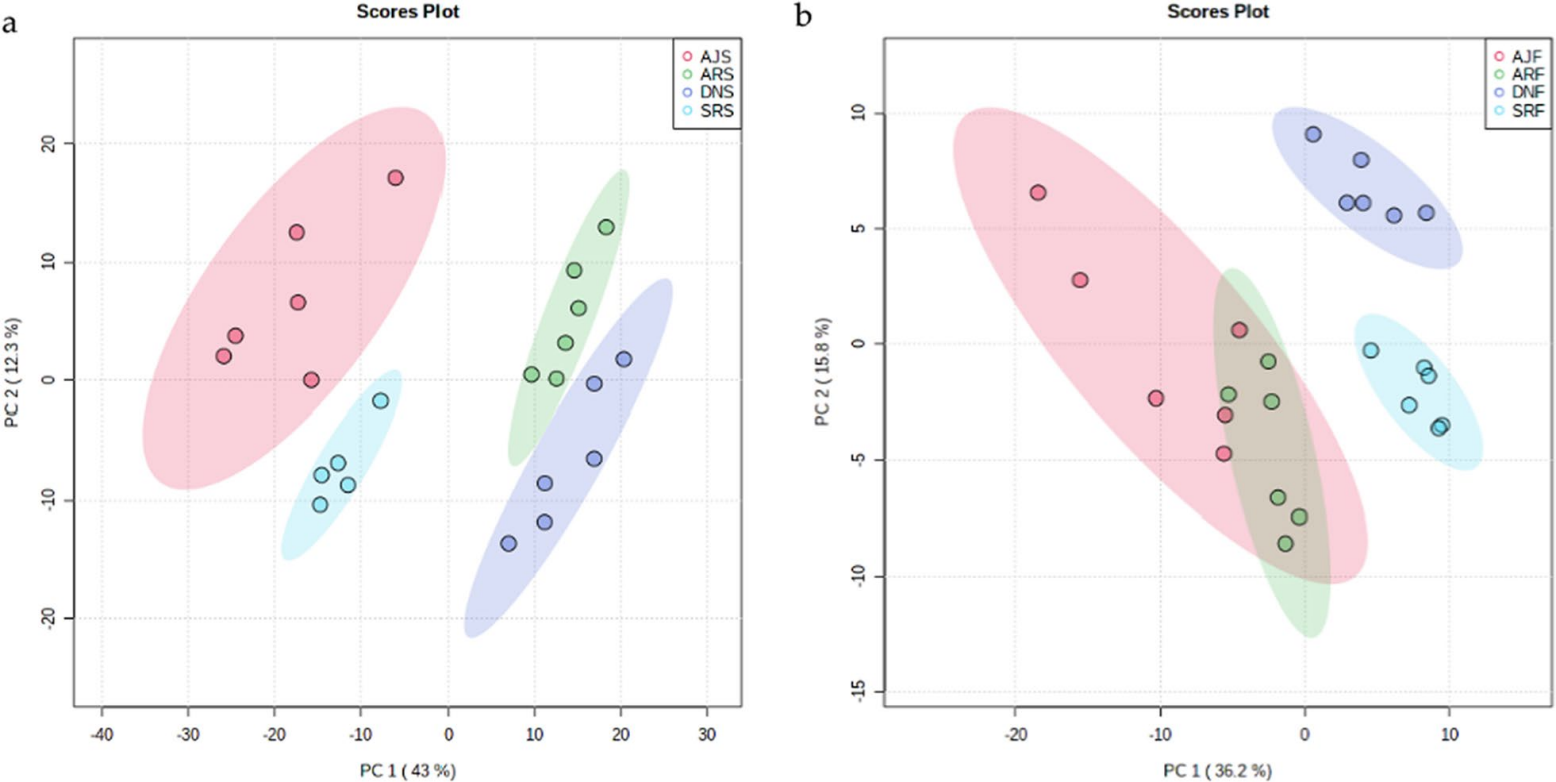
PCA of the four date palm varieties from the GC–MS dataset. **a** Score plots of seed metabolome clusters (PC1 = 43% and PC2 = 12.3%) and **b** flesh metabolome clusters (PC1 = 36.2% and PC2 = 15.8%). In **a**, one replicate of the SRS group was excluded from the PCA analysis

Statistical tools were applied to further investigate the derived volatile compounds identified by GC–MS. PCA score plots were generated using the GC–MS data for all samples (Fig. 8) to probe the differences and similarities in identified metabolites among the sub-groups of samples. As shown in Fig. 8a, the identified metabolites among all seed samples were slightly separated, while flesh samples showed overlap for the Ajwa and Anbra samples (Fig. 8b). However, in the Fig. 8b, the sum of PC1 (36.2%) and PC2 (15.8%) is 52% which is more than 50%, so this model explain more than 50% of the variables.

The significant loadings were confirmed using Kruskal–Wallis ANOVA based on an adjusted *P*-value (FDR) cutoff of 0.05 (Additional file 1: Fig. S2); the data are provided in the supplementary materials. The top 40 VIP for the seed samples showed a high abundance of serotonin (C_10_H_12_N_2_O) and glutaric acid C_5_H_8_O_4_ in the Ajwa variety (Fig. 4A). Moreover, the heatmap analysis for both flesh and seed samples clustered together (Ajwa and Anbra) and (Sukkari and Deglet N), which indicated the majority of discriminating molecules were sugars (Additional file 1: Fig. S5).

### NMR analysis

Additional file 1: Fig. S6A shows the similarity in the types and numbers of signals for all spectra in methyl, methylene, and carbohydrates regions from four different types of date seeds. Whereas carbohydrate sensitivity varies, we observed the sensitivity by comparing the date seed spectra with other sucrose signal spectra with a similar signal-to-noise ratio. Additional file 1: Fig. S6B shows that the highest and lowest concentration of sucrose is found in SRS (f) and AJS (h), respectively. Additional file 1: Fig. S6C also shows similarities among all spectra. Additional file 1: Fig. S7A shows more considerable variation in the methyl region by both number of signals and concentration level, but similarity in the methylene region across the four different types of palm date flesh. Additional file 1: Fig S7B shows the highest and lowest concentration of sucrose in SRF (f) and AJF (h), respectively. Additional file 1: Fig. S7C also shows similarities among all spectra.

### Antibacterial activity

The in vitro antibacterial activities of the date extracts towards the Gram-negative bacterium *Escherichia* *coli* (*E.* *coli*) were investigated using the disc diffusion and MIC methods. Antibacterial effects were assessed by measuring the diameter of growth inhibition and determination of the minimum inhibitory concentrations (MIC), as listed in Table 3. In general, the seed extracts displayed stronger inhibitory effects than the flesh extracts against the tested bacterial strain with zones of inhibition in the range of 18-27 mm. Among all the seed extracts, Ajwa seeds showed the highest antibacterial activities of the tested date varieties, with a zone of inhibition of 27 mm against *E.* *coli* (Additional file 1: Fig. S8). The MIC showed that Ajwa seed extracts inhibited bacterial growth at 20 μM – the closest value to the standard drug (Ampicillin, 12 μM). In general, however, the seed extracts had lower MIC than the flesh extracts.

**Table 3:** Antimicrobial activity of different date extracts based on inhibition zone diameters and MIC

Sample	Inhibition zones (mm)	Minimum inhibition concentration (μM)
Ajwa Flesh	20 ± 0.15	28
Deglet N Flesh	23 ± 01.50	27
Sukkari Flesh	11 ± 1.50	35
Anbra Flesh	14 ± 1.50	32
Ajwa Seed	27 ± 0.05	20
Deglet N Seed	24 ± 0.05	25
Sukkari Seed	11 ± 1.50	32
Anbra Seed	18 ± 0.15	30
Ampicillin	35 ± 0.05	12

### Antioxidant activity

Seed extracts had stronger ABTS radical-scavenging activity than flesh extracts. As shown in Table 4, Ajwa seeds had the highest antioxidant effect (with lowest IC_50_; 54 µg/mL compared to ascorbic acid; 33 µg/m), possibly due to the high concentrations of hydroxyadipic acid and ascorbic acid detected by UHPLC-MS.

**Table 4:** Antioxidant activity of date extracts

Sample	IC_50_μg /mL
Ajwa Flesh	719 ± 1
Deglet N Flesh	946 ± 5.51
Sukkari Flesh	479 ± 0.58
Anbra Flesh	1004 ± 0.05
Ajwa Seed	54 ± 3.61
Deglet N Seed	449 ± 2.31
Sukkari Seed	258 ± 0.58
Anbra Seed	99 ± 1.53
Ascorbic acid	31.5 ± 0.05

IC_50_, determined by applying different concentrations from each extract using the ABTS• + assay

## Discussion

Glucose and fructose are the predominant monosaccharides in data flesh, followed by sucrose [45, 46]. The high abundance of glucose and fructose in flesh samples among all cultivars (Additional file 1: Tables S2 and S4) confirms their use as rapid energy sources, as these sugars are easily absorbed without digestion from the small intestine [47]. NMR confirmed the MS data in terms of carbohydrates as availability of glucose followed by sucrose in flesh samples as shown in Additional file 1: Fig S7. It has been reported that date fruits are very rich in polyphenols [8, 45], and that date seeds are especially rich in phenolic antioxidants and flavonoids [8, 48]. Similarly, we found that the date fruits and seeds vary in terms of nutritional and phytochemical composition. Overall, these nutritional phytochemical compound variations in flesh and seeds of different varieties could be explained by physiological and environmental factors [10, 19]. For instance, the maturity stage, date type, environmental stress (e.g., drought, salinity, temperature, insect pests, and disease), and geographical region could affect date quality [10, 19].

Polyphenols are a significant class of plant secondary metabolites, having polyphenolic structures and which are characterized by their biological activities including antioxidant properties [49, 50]. In this study, we found molecules of phenolic acids in both seed and flesh, such as 4-hydroxybenzoic acid, which has previously been reported for the mesocarp tissue extracts of sugar date palm [*Phoenix* *sylvestris* (L.) Roxb.] [51]. In addition, gentisic acid, which was only found in seeds, is considered an aspirin metabolite and is found naturally in the roots of the genus *Gentiana* and in the fungus *penicillium* *brevicompactum* [52]. Aspirin is known to be metabolized into gentisic acid, and is commonly used to relieve pain, fever, inflammation and prevent heart attack and stroke [52]. Furthermore, gentisic acid has been shown to have antioxidant activity against oxidative stress produced chemically, enzymatically (by myeloperoxidase), and by UV radiation [52, 53]. Another detected metabolite was vanillic acid, which is a flavoring agent and oxidized form of vanillin [54]. Vanillic acid has anti-inflammatory effects, suppressing the production of proinflammatory cytokines, such as lipopolysaccharide [LPS])-induced tumor necrosis factor-alpha (TNF-α), interleukin (IL)-6, and the activation of nuclear factor-kappa B (NF-κB) and caspase-1 [54].

Another sub-class of phenolic acids found by our analysis in flesh and seeds of dates was hydroxycinnamic acids and their derivatives, such as caffeic acid. Caffeic acid (3,4-dihydroxycinnamic acid), the most important class of the hydroxycinnamic acid group required in the human diet as we do not produce these acids ourselves, has been explored as an antioxidant, anti-inflammatory, and antibacterial agent [55–57]. Ferulic acid and sinapic acid were only found in flesh samples. Sinapic acid (3,5-dimethoxy-4-hydroxycinnamic acid) is a common dietary component widespread in cereals, fruits, and vegetables. Its derivatives have substantial bioactivities; for instance, 4-vinylsyringol (a sinapic acid derivative product) has significant antioxidant and anticancer properties and inhibits carcinogenesis and the generation of inflammatory cytokines [58].

Flavonoids are well known for their health-promoting properties and are currently considered to be a vital ingredient in a wide range of nutraceutical, pharmaceutical, medical, and cosmetic products [50]. For example, the presence of catechins (C_15_H_14_O_6_) in seeds only could perhaps explain their known function against DNA damage within plant cells [59, 60]. We also detected procyanidin B2 in seeds, which is a potent anti-diabetic agent [61].

Flavanols are a sub-class of flavonoids that are commonly present in dates [45], and in line with this, for example, (−)-epicatechin (C1_5_H_14_O_6_) was detected in our seed analysis. It is reported that consuming modest amounts of cacao products, such as cocoa, which are rich in the flavanol (−)-epicatechin, significantly decreases cardiometabolic risks [62].

Additionally, (−)-epicatechin might partially reverse the deleterious effects of aging on muscle growth and differentiation [62]. Some vitamins were also detected, like inositol (myo-inositol), a sugar and member of the B vitamin family, which was found relatively abundantly in date seeds compared to flesh, with the highest inositol abundance detected in the Ajwa seeds. Furthermore, we found that date flesh contains much lower oil levels than date seeds, similarly to previous reported values of levels of oil, ranging from 0.2% to 0.5%, compared to 9.7% in seeds [8], suggesting the potential of date seeds as a potent oil source.

In addition, date palm cultivars are known to accumulate organic acids, such as citric acid and malic acid, during ripening to avoid spoilage, suggesting that these organic acid metabolites may contribute to preserving dates for a long time [45]. In our study, citric acid and malic acid were the predominant organic acids identified in the seed and flesh compositions, with high levels specifically in Ajwa seed and Deglet N flesh. This finding could indicate that date fruits and seeds may have antibacterial characteristics and properties, a finding supported by the results of the antibacterial assay. We also found metyrosine (alpha-methyl-L-tyrosine), a selective tyrosine hydroxylase enzyme inhibitor and a known drug with antihypertensive, anti-inflammatory, and anti-ulcerative effects, in both date flesh and seed profiles using UHPLC-MS analysis [63].

The presence of a high abundance of gamma aminobutyric acid (GABA) in Ajwa seeds may be a native component of the cultivar which protect the plant against environmental stress. Notably, GABA acts as an inhibitory neurotransmitter in mammals and protects plants against environmental stress [64]. Overall, considering the detected findings of nutritious and polyphenol-rich compounds in date fruits and seeds via UHPLC–MS and GC–MS, our findings support and shed light on the age old experience of health benefits from regular intake of dates [6, 65].

Based on PLS-DA and PCA of the metabolic profiling, the PLS-DA of UHPLC-MS and PCA of GC–MS datasets demonstrated segregation among all date samples (flesh and seed), with each date type clearly segregated (Figs. 2, 8) by similarities and differences in their metabolite compositions (e.g., sugar content, polyphenols, and fatty acids). Indeed, the heatmap analysis shown above, illustrate how two types of dates were clustered together, namely Ajwa with Anbra and Deglet N with Sukkari (Fig. 4). However, the similarities and differences observed in the metabolite compositions could be explained by fundamental differences in various metabolic responses of the date plants to environmental conditions. Indeed, genotypes and geographical regions have been shown to affect the metabolite profiles of many crops [66]. Therefore, we believe that the clustering could be determined by one (or more) factors, especially as Ajwa and Anbra have the same origin (Medina, Saudi Arabia) and Sukkari and Deglet N have different origins (“Hail, Qassim”, Saudi Arabia and Tunisia, respectively).

Based on the antibacterial and antioxidant activity assays, seed extracts showed stronger ABTS radical-scavenging activity and a lower MIC than flesh extracts. Ajwa seed extract showed the lowest MIC (20 μM) and strongest ABTS radical-scavenging activity (IC_50_: 54 ± 3.61 μg/mL) of the tested seed extracts, possibly due to high concentrations of hydroxyadipic acid and ascorbic acid detected by UHPLC-MS [67–69].

In the flesh analysis, the Deglet N extract (MIC: 27 μM) and Sukkari extract (IC_50_: 479 ± 0.58 μg/mL) showed better anti-bacterial/oxidant activity among other flesh extracts. Moreover, the mixture of polyphenols detected using mass spectrometric have ability to scavenge peroxyl radicals in agreement with the reported potent antioxidant activity of date seed extracts. Thus, we may assume that plant phenolic acids are essential dietary supplements due to their high antioxidant activity and other health benefits [49], and given the identified abundance of some of these compounds in dates, our study support using dates as a staple source of these compounds [70].

In conclusion, our study of the flesh and seeds of the fruits of four date palm cultivars from different countries and regions provides a foundation for the development of more sophisticated in vitro antibacterial models and further investigation of the specific molecules that are responsible for the antioxidative and anti-bacterial activity and other known and perhaps still unknown health benefits of dates.

## Conclusion and future perspectives

The date palm (*Phoenix* *dactylifera* L.) is the most popular fruit in the Middle East and North Africa, where it is frequently consumed and celebrated for its beneficial properties and where it has long since also been and is still considered to be an effective medicine against many ailments. Using laboratory- and instrument-based protocols, the present study highlights the potential of date fruits and their seeds as a low-cost source of not only nutritious food (fruit) or fodder (seed), but also the manufacture of nutritional supplements and pharmaceutical compounds.

The untargeted MS and NMR-based metabolomics approach, coupled with multivariate statistical analysis, reveals that the AJ, AN, SR, and DN date cultivars have distinct metabolite compositions; distinct profiles that likely relate to different genotypes and environmental conditions. Glucose and fructose are the predominant monosaccharides in date flesh, followed by sucrose [45, 46]. The high abundance of glucose and fructose in flesh samples among all cultivars confirms their use as rapid energy sources, as these sugars are easily absorbed without digestion from the small intestine [47]. NMR confirmed the MS data in terms of carbohydrates as availability of glucose followed by sucrose in flesh samples. Moreover, using UHPLC-MS we have shown that date flesh and seed profiles contain several classes of flavonoids, phenolic acids, and amino acid derivatives, including citric acid, malic acid, lactic acid, and hydroxyadipic acid. Additionally, GC–MS profiling showed that date seeds are richer in metabolite classes, such as hydrocinnamic acids (caffeic, ferulic and sinapic acids) than flesh samples.

Ultimately, based on the antibacterial and antioxidant activity assays, Deglet N fruit extract (minimum inhibitory concentration: 27 MIC/μM) and Sukkari fruit extract (IC_50_: 479 ± 0.58 μg /mL) may have higher levels of antibacterial and antioxidative activity than Ajwa and Anbara fruits. However, date seed extracts show lower MIC and stronger ABTS radical-scavenging activity than flesh extracts, thus data seeds may provide a viable and reliable source of antioxidants for the food, fodder, nutritional supplement, and pharmaceutical industries. Specifically, the Ajwa extract shows the best MIC and strongest ABTS radical-scavenging activity among examined seed extracts (MIC: 20 μM; IC_50_: 54 ± 3.61 μg /mL). The different effects may reflect different levels of active compounds with strong free radical scavenging and inhibition properties including phenolic derivatives and flavonoids [71, 72], findings that need further exploration.

## Materials and methods

### Plant material

Five biological replicates of four date varieties at the Tamar stage were collected from different local markets in Saudi Arabia and Tunisia. Ajwa and Anbra dates were obtained from local farms in Medina, Saudi Arabia, and Sukkari dates from Qassim and Hail, Saudi Arabia. Deglet Nour dates were purchased from local farms in Tozeur, Tunisia. These four date varieties have varied origins and post-harvesting conditions. The fruit flesh (F) and seeds (S) of the dates were separately washed from impurities, and lyophilized (Labconco Freezone 4.5) for two days, before finally being ground into fine powder using a freezer/mill grinder.

### Metabolite extraction

Following the protocol from [73], the extraction of metabolites from the polar layer was carried out using methanol, methyl tert-butyl ether (MTBE), and LC-grade water (1:3:3, *v/v/v*). Two solvent extractions (ES) were prepared at a 3:1 (*v/v*) ratio: SE1 (3:1, MTBE:methanol) and SE2 (3:1, LC-grade water:methanol). Aliquots (50µL) of the polar samples were dried (Labconco CentriVap Benchtop Vacuum Concentrators) and stored at -20 °C for the MS and NMR analysis.

### MS and NMR untargeted metabolomics analysis:

#### Chemicals and reagents

12-[[(cyclohexyl-am[42]ino) carbonyl] amino]-dodecanoic acid (CUDA) at a concentration of 50 ppb was used as an internal standard for the UHPLC-MS analysis. For the GC–MS analysis, the derivatization reagent BSTFA (O-bis (trimethyl-silyl) trifluoroacetamide, Sigma-Aldrich, USA) was used. The methoxy-amine (MOX) reagent was 2% methoxy-amine-HCl in pyridine (Sigma-Aldrich, USA). For the NMR analysis, trimethyl-silyl-propane-sulfonic acid (DSS) was used as reference, and deuterium oxide (D_2_O) as a solvent.

#### Sample preparation for MS and NMR

For the UHPLC-MS analysis, 50µL of seed and flesh extracts was dissolved in CUDA/LC-grade water at 3:1 (*v/v*) solution. Pooled samples were prepared by mixing 10µL of each of the five replicates for each date variety. For GC–MS preparation, blank SE solvents and instrument sensitivity check solutions were prepared in addition to the samples. These sensitivity check mixtures consisted of different concentrations (0.1 to 10 ppm) of vacuum-dried carbohydrates and amino acids (alanine, valine, serine, aspartic acid, glutamic acid, α-ketoglutaric acid, asparagine, and glucose). Each sample (50µL) was re-suspended in 30 μl MOX and incubated for 90 min at 30 °C. For the derivatization step in the GC–MS analysis, the samples were derivatized in 100μL BSTFA for 30 min at 37 °C. The BSTFA solution was spiked with a mixture of straight-chain hydrocarbons (HCs) of different lengths (CH7–CH40) to determine the Kovats retention indices.

For the NMR analysis, 50µL of pellets was dissolved in 400μL of D2O and 200μL of potassium phosphate buffer (K + buffer) of concentration 20 mM, and pH 7.4. The samples were vortexed till completely dissolved, then centrifuged at 13,000 rpm for 5 min. 500μL was transferred into 5 mm NMR tubes.

#### UHPLC-MS parameters

A liquid chromatography quadrupole time of flight mass spectrometry (LC-QTOF-MS/MS) equipped with electrospray ionization source (ESI) was used for the LC–MS analysis. The mass spectrometric analyses were performed using a Compact II ESI/QTOF/MS (Bruker Daltonics, Bremen, Germany) using the manufacturer’s default settings for detection of small metabolites. All samples were analyzed in positive and negative mode separately to detect the maximum number of metabolites. The temperature of the ESI source was set at 220 °C, dry gas at 9.0L/min, and the nebulizer pressure at 1.8 Bar. The capillary voltage was set at 4500 V and the end plate offset at 500 V. The full scan range was set from 50 to 1300 Da with auto Tandem mass spectrometry (MS/MS) using the manufacturer’s default settings for small molecules. The auto MSMS threshold per 1000 sum was set to 400 counts with 3X the number of precursors and exclusion after three spectra. The spectral rate was set to 12 Hz with a fixed ms/ms acquisition rate of 2 Hz. The QTOF instrument was calibrated before the experiment by directly infusing 10 mM sodium formate solution. In addition, during analysis of every sample in both positive and negative mode, the instrument was calibrated externally using the same sodium formate solution at the beginning of sample injection and internally by using a mixture of three lock mass compounds infused directly into the MS from the source reservoir (these compounds were C_5_H_4_N_4_, C_12_H_18_F_12_N_3_O_6_P_3_, and C_18_H_19_O_6_N_3_P_3_F_24_ with accurate masses of 121.043, 621.022, and 921.002, respectively). The mass accuracy of the instrument was found to be less than 0.5 ppm. Samples (5μL) were separated using a 1.7 μm × 2.1 mm x 100 mm Acquity UHPLC CSH C18 column (Waters, USA) on a UHPLC system (UltiMate 3000, Thermo Scientific, Germany). For UHPLC, mobile phase A was 0.1% formic acid (LC–MS-grade) in water, and mobile phase B was 0.1% formic acid in acetonitrile [73]. Separation was performed at a constant flow rate of 0.4 mL/min and the gradient program was 0% of solvent B at 0.01 min, ramped to 100% B over 10 min, reduced to 0% B at 12 min, then the column was equilibrated with 0% B to 14 min. The column temperature was set at 40 °C and the temperature of the autosampler was kept at 4 °C.

#### UHPLC-MS data analysis

MetaboScape 2022 software (Bruker, Bremen, Germany) was used to analyze the raw MS and MS–MS data. The metabolites were annotated according to the exact mass of the molecules, their molecular formula, and their patterns of tandem mass spectrometry (MS/MS) fragmentation with regards to available libraries such as Bruker Plant Metabolites and Mona Export LC–MS-MS positive and negative modes. The metabolite annotation sources included the human metabolome database (HMDB) metabolites and Bruker Sumner MetaboBASE® Plant Libraries. To validate the instrument method and reproducibility, the RSD% of CUDA (the internal standard in LC) was 11.63% for all flesh samples and 26.20% for seed samples.

#### GC–MS parameters

A single quadrupole GC–MS system (Agilent 7890 GC/5975C MSD) coupled to an EI source with an ionization energy of 70 eV was used to analyze 1µL of the derivatized solutions. The ion source and mass analyzer temperatures were kept at 230 °C and 150 °C, respectively, with a solvent delay of 8.0 min.

The auto-tuning of the mass analyzer was set according to the manufacturer’s instructions, and the scan was fixed from 35–700 Da with a scan speed 2 scans/s. A DB-5MS fused silica capillary column (30 m × 0.25 mm I.D., 0.25 µm film thickness; Agilent J&W Scientific, Folsom, CA) was utilized for chromatographic separation, which was chemically coupled with a 5% phenyl methyl-poly-siloxane cross-linked stationary phase. Helium was utilized as the carrier gas at a constant flow rate of 1.0 mL/min^−1^. Samples were injected (1μL) in random order into a 5977A gas chromatograph (Agilent, USA). Metabolites were separated using a 30 m × 250 μm × 0.25 μm column (TR-5MS Thermo, USA). The oven program was set to 70 °C for 4 min, then ramped at a rate of 6 °C/min to 330 °C with a 5-min hold time. The GC inlet temperature was set at 250 °C, and the temperature of the transfer line to the MS EI source was kept at 320 °C. The derivatized solution of each sample (1µL) was injected into a split and split-less inlet using an autosampler equipped with a 10µL syringe. The GC inlet was operated in split mode to analyze the date flesh samples and in splitless mode for the date seed samples. The total run time was 52.3 min.

#### GC–MS data analysis and compound identification

For GC–MS data processing, the NIST 14 database was used to identify metabolites in all samples using Agilent MSD Chemstation software. MS-DIAL software (v. 4.38) was used to analyze the.abf files after installing the library (all records with Kovats RI) from the MS-DIAL website [74]. The default MS-DIAL parameters were applied, with the exception that the scan range was 35 to 700 Da and blank subtraction with a sample/blank ion ratio less than threefold was applied. The pool mixed sample was used for the peak alignment. Metabolites were identified in MS–DIAL 4.62 using MS–DIAL (‘GCMS DB-Public-KovatsRI-VS3.msp’ spectra library; available free at http://prime.psc.riken.jp/compms/msdial/main.html) with carbohydrate mix as the retention time (RT) index and by comparison with the Kovats retention indices (RIs) within the library. For validation of the instrument method and reproducibility, the RSD% of four HCs (the internal standard set GC) was calculated and found to be 28.23% for all flesh samples and 28.72% for all seed samples.

#### NMR analysis

A Bruker 800 MHz AVANACE NEO NMR spectrometer equipped with Bruker TCI (2 H/ 13 C/ 15 N) cryogenic probe (BrukerBioSpin, Rheinstetten, Germany) was used to record all NMR spectra. Noesypr1d for 1D 1H NMR spectra were applied to collect the data [75]. Data were collected at 298 K for 96 scans with a 5 s inter-scan delay and a 2 s per-scan acquisition time. The total acquisition time for each sample was about 15 min. Topspin (4.1.4) was used for spectral processing following the acquisition. All spectra were zero-filled to 131 k points, and a 0.3 Hz line broadening was used.

### Multivariate data analysis

The web-based platform MetaboAnalyst 5.0 (https://www.metaboanalyst.ca/) was used to perform partial least squares discriminant analysis (PLS-DA), principal component analysis (PCA) and other statistical analyses such as hierarchical cluster analysis (HCA) [76]. PLS-DA and PCA analysis of the UHPLC-MS and GC–MS datasets was conducted for an average of five biological replicates of the same type of *P.* *dactylifera*. All files were uploaded as comma separated values (.csv) and the samples were assessed in unpaired columns.

Prior to performing statistical analysis, the data was sum-normalized and the intensities of the signals of the identified metabolites were log-transformed and auto-scaled (mean-centered and divided by the standard deviation of each variable) over the samples.

### Validation of biological activity

#### In vitro antibacterial activity

The antibacterial activity of the extracts against *Escherichia* *coli* (ATCC: 25,922) was evaluated using the agar disc diffusion and broth dilution methods [77]. The bacterial strain was cultured on Muller–Hinton agar (MHA) plates for 18–24 h at 35 °C.

#### Disc diffusion method

The extracts were prepared by dissolving 20 mg of each compound in 1 mL of DMSO. DMSO was used as a negative control for all samples. Ampicillin was used as a positive control drug for significant antibacterial activity. Bacterial cultures adjusted to McFarland 0.5 standard solution (1.5 × 10^8^ CFU/mL), were spread onto Hinton agar plates using a sterile swab. Paper discs of 5 mm diameter were impregnated individually with 100 μg/mL of the samples and placed on the plates. Plates were incubated at 37 °C for 18–24 h and the antibacterial activity of each test sample was determined by measuring the diameter of the zone of inhibition and comparing it with the zone of inhibition for the positive control drug. The antibacterial behavior of each test sample was measured three times. No inhibition zone was observed for the DMSO negative control.

#### Determination of minimal inhibitory concentration (MIC)

The antibacterial activities of all extracts and standard drugs were also evaluated using the minimal inhibitory concentration (MIC) method. Each extracted sample and standard drug were individually prepared in DMSO to obtain 2000 µg/mL concentration (stock solution). The aim of this method was to find the lowest concentration of the examined antibacterial agent that inhibits visible growth of the microorganism being investigated. Luria–Bertani (LB) broth was used as bacterial McFarland standard solution for each complex tested for antibacterial activity and the standard drug (ampicillin). Thirty-nine tubes of 5 mL volume were used in five rows such that each row contained 13 tubes. Each row represents the tested extracts. Afterwards, 1 mL LB broth (rows 1–4) and 1 mL standard drug (ampicillin) broth (row 5) were added in tubes 1–13 in each row. Then, 1 mL of tested compound (stock solution) was added to the first tube in each row and mixed. After mixing, 1 mL of the first tube in each row was serially carried over to the second tube in the same row and mixed, and the content of the second tube was transferred to the third tube in each row. This serial dilution was repeated until tube 12 in each row, and1 mL of tube 12 was discarded. Tube 12 had no bacteria and was used as a negative control. Tube 13, without antibacterial agent, was used as a positive control. Thus, the micro-dilution provided antibacterial concentrations of 1000, 500, 250, 125, 62.5, 31.25, 15.62, 7.81, 3.90, 1.95, 0.975, and 0.487 µg/mL, respectively. Finally, 100µL bacterial suspension was added to tubes 1–11 and 13 in rows 1–5, and the tubes were incubated for 24 h at 37 °C. The highest dilution of active sample to inhibit evident growth of the microorganism was expressed as the MIC.

#### Antioxidant assay

The ABTS^*+^ radical-scavenging activity of the date extracts was determined according to [78]. Briefly, 2,2'-azinobis-[3-ethylbenzthiazoline-6-sulfonic acid] (ABTS) was dissolved in twice-distilled water at 7mM. ABTS radical cation solution was prepared by reacting the ABTS solution with K_2_S_2_O_8_ solution (final concentration: 2.45 mM) for 12–16 h in the dark at room temperature. To determine the antioxidant capacity of the extracts, ascorbic acid was used as a standard, and the ABTS solution was diluted with an appropriate solvent (methanol) to an absorbance of 0.7 ± 0.01. The ABTS radicals were prepared freshly each time according to the original method. A 10 µL of standard or extracts was added to 190 µL of ABTS reagent in 96 well plate. The plate was shaken for 10 s at medium speed, following 5 min incubation in the dark and absorbance was measured at 734 nm. The samples and reference (ascorbic acid) were tested at different concentrations (1, 3, 5, and 10 µg/mL). All measurements were carried out at least three times. Percent inhibition of absorbance at 734 nm was calculated using the following formula:

ABTS· + scavenging effect (%) = ((A_control_–A_sample_)/ A_control_) × 100.

### Supplementary Information

**Additional file 1: Table S1. **Metabolites detected in the seeds of four P. dactylifera varieties (AJS, ARS, SRS, DNS) by LC-MS (merged positive and negative ionization data). Different classes of metabolites are present, including amino acids, fatty acids, carbohydrates, vitamins and polyphenol compounds. The resulting 98 known metabolites are shown. **Table S2. **Identified metabolites of four dates fruits (AJF, ARF, SRF, DNF) using the merged LC-MS data. Containing different classes of metabolites, including amino acids, fatty acids, carbohydrates, vitamins, and polyphenols compounds. Resulting of 96 known metabolites. **Table S3.** Metabolite profiling of seed samples of four P. dactylifera varieties (AJS, ARS, SRS, DNS) using GC/MS data. Containing different amino acids, fatty acids, carbohydrates, polyphenols compounds. **Table S4.** Metabolomic profiling of all four P. dactylifera cultivars of flesh samples of GC/MS data. Showing different classes of metabolites include amino acids, carbohydrates. **Table S5. **Significance analysis (p<0.05 and FDR) of date seed 40VIP scores (of all known and unknown compounds); the LC MS data. **Table S6. **Significance analysis (p<0.05 and FDR) of date flesh 40VIP scores (of all known and unknown compounds), the LC MS data. **Table S7. **Significance analysis (p<0.05 and FDR) of date seed 40VIP Scores (of all known compounds only), the LC MS data. **Table S8. **Significance analysis (p<0.05 and FDR) of date flesh 40VIP Scores (of all known compounds only), the LC MS data. **Figure S1.** One-way ANOVA test of UHPLC-MS data, illustrating the a) 525 significant metabolites (red circles) identified in seed samples, and b) 686 significant molecules in the flesh samples. **Figure S2.** One-way ANOVA test of GC-MS data, illustrating the a) 173 significant metabolites (red circles) identified in seed samples, and b) 128 significant met in flesh samples. **Figure S3.** Heatmap visualization of 40 VIP-annotated compounds; (a) Heatmap representation of 40 VIP-annotated compounds from the flesh profiling analysis and (b) from the seed analysis. **Figure S4.** 40 VIP metabolites that significantly associate with date varieties segregation (GC-MS). a. seed, b. flesh. **Figure S5.** HCA of the four date cultivars based on GC-MS data using MetaboAnalyst software. The heatmap shows date varieties a) seed samples, and b) flesh samples. **Figure S6.** (A), (B), and (C) Display toggled expanded 1D 1H NMR spectra from the regions 0.78 to 4.61 ppm, 4.65 to 5.42 ppm, and 5.09 to 8.48 ppm, respectively of different palm date seeds as (a, e, p) for DNS, (b, f, q) for SRS, (c, g, r) for ARS, and (d, h, s) for AJS recorded by using solution-state NMR of 800 MHz in D2O solution. **Figure S7.** (A), (B), and (C) Display toggled expanded 1D 1H NMR spectra from the regions 0.79 to 4.62 ppm, 4.60 to 5.43 ppm, and 5.09 to 8.49 ppm, respectively of different palm date flesh as (a, e, p) for DNF, (b, f, q) for SRF, (c, g, r) for ARF, and (d, h, s) for AJF recorded by using solution-state NMR of 800 MHz in D2O solution. **Figure S8.** Antibacterial activity of different seed extracts with gram-negative bacteria (E. coli). DMSO was used as a negative control. Examples of inhibition zones in MH- agar plates for different date seed methanol extracts.

## Data Availability

The GC–MS data and LC–MS data were deposited in the MetaboLights database (https://www.ebi.ac.uk/metabolights/) as: 1—for the LCMS data: MTBLS5988; 2—for the GCMS data: MTBLS6437; Both are validated, and in curation.
