# Supplementary material for: Untargeted metabolomics analysis of four date palm (Phoenix dactylifera L.) cultivars using MS and NMR
Source: Nat Prod Bioprospect. 2023 Oct 23;13(1):44. doi: 10.1007/s13659-023-00406-y (PMC10593664; doi:10.1007/s13659-023-00406-y)
Supplement: Supplementary file 1 — Additional file 1: Table S1. Metabolites detected in the seeds of four P. dactylifera varieties (AJS, ARS, SRS, DNS) by LC-MS (merged positive and negative ionization data). Different classes of metabolites are present, including amino acids, fatty acids, carbohydrates, vitamins and polyphenol compounds. The resulting 98 known metabolites are shown. Table S2. Identified metabolites of four dates fruits (AJF, ARF, SRF, DNF) using the merged LC-MS data. Containing different classes of metabolites, including amino acids, fatty acids, carbohydrates, vitamins, and polyphenols compounds. Resulting of 96 known metabolites. Table S3. Metabolite profiling of seed samples of four P. dactylifera varieties (AJS, ARS, SRS, DNS) using GC/MS data. Containing different amino acids, fatty acids, carbohydrates, polyphenols compounds. Table S4. Metabolomic profiling of all four P. dactylifera cultivars of flesh samples of GC/MS data. Showing different classes of metabolites include amino acids, carbohydrates. Table S5. Significance analysis (p<0.05 and FDR) of date seed 40VIP scores (of all known and unknown compounds); the LC MS data. Table S6. Significance analysis (p<0.05 and FDR) of date flesh 40VIP scores (of all known and unknown compounds), the LC MS data. Table S7. Significance analysis (p<0.05 and FDR) of date seed 40VIP Scores (of all known compounds only), the LC MS data. Table S8. Significance analysis (p<0.05 and FDR) of date flesh 40VIP Scores (of all known compounds only), the LC MS data. Figure S1. One-way ANOVA test of UHPLC-MS data, illustrating the a) 525 significant metabolites (red circles) identified in seed samples, and b) 686 significant molecules in the flesh samples. Figure S2. One-way ANOVA test of GC-MS data, illustrating the a) 173 significant metabolites (red circles) identified in seed samples, and b) 128 significant met in flesh samples. Figure S3. Heatmap visualization of 40 VIP-annotated compounds; (a) Heatmap representation of 40 VIP-annotated compou [file 13659_2023_406_MOESM1_ESM.docx]

### Additional Materials

**Untargeted Metabolomics of Four Date Palm (*Phoenix dactylifera* L.) Cultivars Using MS and NMR**

**Shuruq Alsuhaymi^1^, Upendra Singh^1^, Inas Al-Younis^1^, Najeh M. Kharbatia^2^, Ali Haneef^3^, Kousik Chandra^1^, Manel Dhahri^4^, Mohammed A. Assiri^5^, Abdul-Hamid Emwas^2*^ and Mariusz Jaremko^1*^.**

^1^ Biological and Environmental Science and Engineering Division, King Abdullah University of Science and Technology (KAUST).

^2^ Core Labs, King Abdullah University of Science and Technology (KAUST).

^3^ King Abdullah International Medical Research Center (KAIMRC).

^4^ Biology Department, Faculty of Science, Taibah University, Yanbu Branch, Saudi Arabia

^5^ Department of Pharmacology and Toxicology, College of Pharmacy, King Saud University, Riyadh, Saudi Arabia

**Table S1.** Metabolites detected in the seeds of four *P. dactylifera* varieties (AJS, ARS, SRS, DNS) by LC-MS (merged positive and negative ionization data). Different classes of metabolites are present, including amino acids, fatty acids, carbohydrates, vitamins and polyphenol compounds. The resulting 98 known metabolites are shown.

| **RT (min)** | **m/z** | **Metabolite Name** | **Formula** |
| --- | --- | --- | --- |
| 0.52 | 175.11904 | Arginine | C6H14N4O2 |
| 0.53 | 104.07067 | gamma-Amino-n-butyric acid | C4H9NO2 |
| 0.64 | 133.06059 | L-Asparagine | C4H8N2O3 |
| 0.66 | 130.05014 | L-Pyroglutamic acid | C5H7NO3 |
| 0.66 | 176.10318 | L-Citrulline | C6H13N3O3 |
| 0.68 | 148.06079 | Glutamic Acid | C5H9NO4 |
| 0.69 | 258.11025 | Glycerophosphocholine | C8H20NO6P |
| 0.71 | 134.04497 | L-Aspartic Acid | C4H7NO4 |
| 0.71 | 116.07068 | L-Proline | C5H9NO2 |
| 0.71 | 179.05613 | D-Galactose | C6H12O6 |
| 0.71 | 181.07092 | Mannitol | C6H14O6 |
| 0.73 | 146.08177 | L-trans-5-Hydroxy-2-piperidinecarboxylic acid | C6H11NO3 |
| 0.77 | 118.08645 | N-Methyl-a-aminoisobutyric acid | C5H11NO2 |
| 0.78 | 365.10574 | Sucrose | C12H22O11 |
| 0.79 | 163.06028 | 3-hydroxymethyl-glutaric acid | C6H10O5 |
| 0.85 | 139.03886 | Salicylic acid | C7H6O3 |
| 0.86 | 132.1021 | DL-Norleucine | C6H13NO2 |
| 0.87 | 135.02979 | Threonic acid | C4H8O5 |
| 0.87 | 195.0511 | Gluconic acid | C6H12O7 |
| 0.89 | 173.04607 | Shikimic acid | C7H10O5 |
| 0.96 | 243.06253 | Uridine | C9H12N2O6 |
| 1.12 | 133.01429 | DL-Malic acid | C4H6O5 |
| 1.17 | 237.06171 | 2-Keto-3-deoxyoctonic acid | C8H14O8 |
| 1.35 | 182.08124 | Tyrosine | C9H11NO3 |
| 1.36 | 160.06045 | 4-methyleneglutamic acid | C6H9NO4 |
| 1.52 | 259.0215 | Glucose-1-phosphate | C6H13O9P |
| 1.82 | 421.07522 | Lactose 6-phosphate | C12H23O14P |
| 2.06 | 147.02913 | 2-Oxoglutaric acid | C5H6O5 |
| 2.07 | 191.01981 | Citric acid | C6H8O7 |
| 2.32 | 142.05107 | Methyl L-pyroglutamate | C6H9NO3 |
| 2.46 | 166.08648 | DL-Phenylalanine | C9H11NO2 |
| 2.76 | 139.03906 | 4-Hydroxybenzoic acid | C7H6O3 |
| 2.77 | 299.0772 | Salicylic acid glucoside | C13H16O8 |
| 2.81 | 343.10385 | 4',6'-dihydroxy-2'-methoxyacetophenone 6'-glucoside | C15H20O9 |
| 2.85 | 203.0827 | L-Tryptophan | C11H12N2O2 |
| 2.85 | 188.07072 | 3-Indoleacrylic acid | C11H9NO2 |
| 2.9 | 315.07249 | Gentisic acid 5-O-glucoside | C13H16O9 |
| 3 | 169.04966 | Vanillic acid | C8H8O4 |
| 3.05 | 153.01926 | Gentisic acid | C7H6O4 |
| 3.06 | 109.02941 | Hydroquinone | C6H6O2 |
| 3.19 | 199.06063 | Syringic acid | C9H10O5 |
| 3.2 | 359.09853 | Benzoic acid + 1O, 2MeO, O-Hex | C15H20O10 |
| 3.29 | 443.19205 | Luteolin 7-glucoside | C21H32O10 |
| 3.32 | 577.13449 | Procyanidin B2 | C30H26O12 |
| 3.32 | 427.10147 | Epiafzelechin 3-O-gallate | C22H18O9 |
| 3.34 | 575.11864 | Procyanidin A1 | C30H24O12 |
| 3.38 | 865.1989 | Procyanidin C1 | C45H38O18 |
| 3.42 | 480.22349 | 1-O-Desmethyltetrabenazine glucuronide | C24H33NO9 |
| 3.43 | 341.0881 | Caffeic acid 3-glucoside | C15H18O9 |
| 3.43 | 181.04985 | Caffeic Acid | C9H8O4 |
| 3.46 | 290.075 | Quinazoline, 4-(2-quinolinylthio) | C17H11N3S |
| 3.47 | 291.08704 | Epicatechin | C15H14O6 |
| 3.48 | 287.05597 | Dihydrokaempferol | C15H12O6 |
| 3.51 | 293.12424 | Ethyl (S)-3-hydroxybutyrate glucoside | C12H22O8 |
| 3.51 | 595.25967 | 2-(2-hydroxyethoxy)ethanol;2-[2-(2-hydroxyethoxy)ethoxy]ethanol;2-methylprop-2-enoate | C26H44O15 |
| 3.52 | 549.2547 | Blumenin | C25H40O13 |
| 3.68 | 112.98558 | Trifluoroacetic acid | C2HF3O2 |
| 3.78 | 335.07727 | 5-O-Caffeoylshikimic acid | C16H16O8 |
| 3.79 | 176.07031 | Indole-3-acetate | C10H9NO2 |
| 3.79 | 333.06148 | Hovenitin I | C16H14O8 |
| 3.79 | 163.0392 | Umbelliferone | C9H6O3 |
| 3.86 | 281.13934 | Dihydrophaseic acid | C15H22O5 |
| 3.9 | 329.08815 | Vanillic acid glucoside | C14H18O9 |
| 4 | 461.16694 | Decaffeoyl-acteoside | C20H30O12 |
| 4.05 | 375.23772 | Tsangane L 3-glucoside | C19H34O7 |
| 4.39 | 247.13314 | Xanthatin | C15H18O3 |
| 4.73 | 461.10875 | Tectoridin | C22H22O11 |
| 5.36 | 263.12927 | Abscisic acid | C15H20O4 |
| 5.79 | 274.27435 | Lauryldiethanolamine | C16H35NO2 |
| 6.01 | 256.26354 | Palmitamide | C16H33NO |
| 6.04 | 152.0354 | 3-Methyl-4-nitrophenol | C7H7NO3 |
| 6.18 | 329.23365 | 9,10,13-TriHOME(11) | C18H34O5 |
| 6.6 | 318.30017 | Phytosphingosine | C18H39NO3 |
| 6.64 | 158.15424 | N-Isobutyl-3-methylbutanamide | C9H19NO |
| 6.98 | 185.0077 | Chelidonic acid | C7H4O6 |
| 7.02 | 199.04353 | 4-(Propan-2-yl)benzenesulfonic acid | C9H12O3S |
| 7.58 | 597.3047 | LPI 18:1 | C27H51O12P |
| 7.89 | 468.30804 | 1-Myristoyl-sn-glycero-3-phosphocholine | C22H46NO7P |
| 7.89 | 194.08237 | Metyrosine | C10H13NO3 |
| 8.32 | 476.27858 | Lyso-PE(18:2) | C23H44NO7P |
| 8.45 | 520.34017 | 1-Linoleoyl-sn-glycero-3-phosphorylcholine | C26H50NO7P |
| 8.79 | 221.15503 | 3,6-Ditert-butyl-1,2-benzenediol | C14H22O2 |
| 8.82 | 496.34006 | 1-Palmitoyl-sn-glycero-3-phosphocholine | C24H50NO7P |
| 9.01 | 478.29319 | PE(18:1(9Z)/0:0) | C23H46NO7P |
| 9.05 | 522.35542 | 1-Oleoyl-sn-glycero-3-phosphocholine | C26H52NO7P |
| 9.94 | 338.34169 | Erucamide | C22H43NO |
| 9.98 | 704.52003 | PC(15:0_15:1) | C38H74NO8P |
| 10.05 | 300.28963 | Palmitoylethanolamide (PEA) | C18H37NO2 |
| 10.06 | 786.59687 | 1,2-Dioleoyl-sn-glycero-3-phosphatidylcholine | C44H84NO8P |
| 10.2 | 285.27871 | Palmitic Acid ethyl ester | C18H36O2 |
| 10.4 | 571.28894 | LPI 16:0 | C25H49O12P |
| 10.6 | 282.2791 | Oleamide | C18H35NO |
| 10.83 | 760.58201 | 2-Oleoyl-1-palmitoyl-sn-glycero-3-phosphocholine. | C42H82NO8P |
| 10.83 | 784.58343 | PC(18:1_18:2 | C44H82NO8P |
| 10.94 | 397.38332 | Stigmastane steroids | C29H48 |
| 10.96 | 621.43782 | Ginsenoside | C36H62O8 |
| 11.14 | 732.55308 | 1-Oleoyl-2-myristoyl-sn-glycero-3-phosphocholine | C40H78NO8P |
| 12.02 | 835.53505 | 1-Hexadecanoyl-2-(9Z-octadecenoyl)-sn-glycero-3-phospho-(1'-myo-inositol) | C43H81O13P |

**Table S2.** Identified metabolites of four dates fruits (AJF, ARF, SRF, DNF) using the merged LC-MS data. Containing different classes of metabolites, including amino acids, fatty acids, carbohydrates, vitamins, and polyphenols compounds. Resulting of 96 known metabolites.

| **RT (min)** | **m/z** | **Metabolite Name** | **Formula** |
| --- | --- | --- | --- |
| 0.54 | 162.0763 | Aminoadipic acid | C6H11NO4 |
| 0.55 | 104.0706 | DL-beta-Aminobutyric acid | C4H9NO2 |
| 0.56 | 175.1186 | Arginine | C6H14N4O2 |
| 0.58 | 267.1268 | 9-(3-sulfanylpropylsulfanyl)nonane-1-thiol | C12H26S3 |
| 0.63 | 133.0607 | L-Asparagine | C4H8N2O3 |
| 0.67 | 220.0813 | O-succinyl-L-homoserine | C8H13NO6 |
| 0.69 | 146.0814 | N-Isobutyrylglycine | C6H11NO3 |
| 0.7 | 136.0623 | Adenine | C5H5N5 |
| 0.73 | 116.0706 | L-Proline | C5H9NO2 |
| 0.76 | 163.0601 | 1,6-Anhydro-.beta.-D-glucose | C6H10O5 |
| 0.77 | 310.1141 | N-Acetylneuraminic Acid | C11H19NO9 |
| 0.78 | 387.1145 | Galactinol . | C12H22O11 |
| 0.78 | 109.0285 | 1,4-Benzoquinone | C6H4O2 |
| 0.78 | 127.039 | Pyrogallol | C6H6O3 |
| 0.81 | 365.1056 | Sucrose | C12H22O11 |
| 0.82 | 161.0457 | 2-Hydroxyadipic acid | C6H10O5 |
| 0.82 | 256.0834 | 4-(purin-6-ylamino)benzoic acid | C12H9N5O2 |
| 0.86 | 315.0937 | 1-[(Dimethoxymethoxy-hydroxy-methoxymethoxy)-hydroxy-methoxymethoxy]-1-methoxyethanol | C10H20O11 |
| 0.86 | 245.066 | 1-tert-butylsulfanylsulfonyl-4-methylbenzene | C11H16O2S2 |
| 0.86 | 195.0511 | Galactonic acid | C6H12O7 |
| 0.86 | 132.102 | Isoleucine | C6H13NO2 |
| 0.87 | 177.102 | Serotonin | C10H12N2O |
| 0.88 | 294.1545 | N-Fructosyl isoleucine | C12H23NO7 |
| 0.88 | 135.0299 | Erythronic acid | C4H8O5 |
| 0.91 | 165.0405 | D-Arabinonic acid | C5H10O6 |
| 0.92 | 503.1621 | Gentianose | C18H32O16 |
| 0.94 | 179.0562 | Aldehydo-D-glucose | C6H12O6 |
| 1 | 203.0525 | Diphenylsulfoxide | C12H10OS |
| 1 | 267.0724 | (2R)-3-Hydroxy-2-(alpha-D-mannopyranosyloxy)propanoic acid | C9H16O9 |
| 1.09 | 475.1307 | Xylosyl-cellobiose | C16H28O16 |
| 1.14 | 237.0618 | 2-Keto-3-deoxyoctonic acid | C8H14O8 |
| 1.17 | 148.0604 | DL-threo-beta-Methylaspartic acid | C5H9NO4 |
| 1.17 | 190.071 | N-Acetyl-L-glutamic acid | C7H11NO5 |
| 1.2 | 191.0658 | Carglumic acid | C6H10N2O5 |
| 1.27 | 341.109 | D-(+)-Trehalose | C12H22O11 |
| 1.34 | 160.0605 | L-trans-alpha-Amino-2-carboxycyclopropaneacetic acid | C6H9NO4 |
| 1.39 | 133.0142 | Malic acid | C4H6O5 |
| 1.42 | 98.98415 | Ortophosphate | H3O4P |
| 1.42 | 176.9359 | Pyrophosphate | H4O7P2 |
| 1.48 | 130.0498 | L-Pyroglutamic acid | C5H7NO3 |
| 1.51 | 117.0194 | Succinic acid | C4H6O4 |
| 1.59 | 204.0863 | Diethyl formamidomalonate | C8H13NO5 |
| 1.62 | 259.0224 | D-Mannose 6-phosphate | C6H13O9P |
| 1.64 | 274.0919 | Pentose + Proline | C11H15NO7 |
| 1.68 | 379.0847 | N-(4-Aminobutyl)-1-[(2R,5R)-2-(phosphonooxymethyl)-1,3-oxathiolane-5-yl]cytosine | C12H21N4O6PS |
| 1.7 | 377.0858 | Bis(1-piperidinecarbodithioic acid)2-hydroxypropane-1,3-diyl ester | C15H26N2OS4 |
| 1.78 | 421.0756 | Lactose 6-phosphate | C12H23O14P |
| 1.87 | 344.1335 | N-Fructosyl tyrosine | C15H21NO8 |
| 2.05 | 191.0198 | Citric acid | C6H8O7 |
| 2.12 | 290.0883 | N-Fructosyl pyroglutamate | C11H17NO8 |
| 2.13 | 147.0303 | Citramalic acid | C5H8O5 |
| 2.24 | 341.1091 | 4.alpha.-Mannobiose | C12H22O11 |
| 2.27 | 268.103 | Adenosine | C10H13N5O4 |
| 2.33 | 611.1435 | Glutathione disulfide | C20H32N6O12S2 |
| 2.42 | 282.0845 | Guanosine | C10H13N5O5 |
| 2.47 | 166.0861 | DL-Phenylalanine. | C9H11NO2 |
| 2.58 | 186.041 | N-Acryloyl-DL-aspartic acid | C7H9NO5 |
| 2.63 | 328.1386 | N-Fructosyl phenylalanine. | C15H21NO7 |
| 2.7 | 408.1495 | Octanoic acid, 2-[[4-chloro-6-[(2,3-dimethylphenyl)amino]-2-pyrimidinyl]thio]- | C20H26ClN3O2S |
| 2.81 | 343.1045 | 3-(3-(.beta.-D-Glucopyranosyloxy)-2-hydroxyphenyl)propanoic acid | C15H20O9 |
| 2.85 | 218.1038 | Pantothenic Acid | C9H17NO5 |
| 2.95 | 367.1499 | Fructose-L-tryptophan | C17H22N2O7 |
| 3.42 | 355.1031 | Feruloyl Hexoside | C16H20O9 |
| 3.44 | 339.1064 | Gerberinside | C16H18O8 |
| 3.46 | 293.1239 | Ethyl (S)-3-hydroxybutyrate glucoside | C12H22O8 |
| 3.47 | 325.0931 | Cis-Melilotoside | C15H18O8 |
| 3.6 | 175.0613 | 2-Isopropylmalic acid | C7H12O5 |
| 3.67 | 112.9856 | Trifluoroacetic acid | C2HF3O2 |
| 3.79 | 337.0909 | 5-O-Caffeoylshikimic acid | C16H16O8 |
| 3.79 | 163.0385 | Umbelliferone | C9H6O3 |
| 3.93 | 181.0494 | Caffeic Acid | C9H8O4 |
| 4.02 | 131.0714 | 2-Ethyl-2-Hydroxybutyric acid | C6H12O3 |
| 4.56 | 193.0508 | Ferulic Acid | C10H10O4 |
| 4.83 | 207.0651 | 7-Hydroxy-4-(methoxymethyl)coumarin | C11H10O4 |
| 4.87 | 209.0803 | 3,5-Dimethoxycinnamic acid | C11H12O5 |
| 4.91 | 183.0777 | Diisopropyl phosphate | C6H15O4P |
| 5.79 | 274.273 | Lauryldiethanolamine | C16H35NO2 |
| 6.04 | 152.0354 | 3-Methyl-4-nitrophenol | C7H7NO3 |
| 6.2 | 329.2333 | Tianshic acid | C18H34O5 |
| 6.61 | 318.2992 | Phytosphingosine | C18H39NO3 |
| 6.64 | 158.1535 | N-Isobutyl-3-methylbutanamide | C9H19NO |
| 6.88 | 253.1448 | Gliocladic acid | C14H22O4 |
| 7.01 | 199.0436 | 4-(Propan-2-yl)benzenesulfonic acid | C9H12O3S |
| 7.89 | 194.08236 | Metyrosine | C10H13NO3 |
| 8.79 | 221.1549 | 3,6-Ditert-butyl-1,2-benzenediol | C14H22O2 |
| 8.84 | 279.2305 | 9Z,11E,13E-Octadecatrienoic acid | C18H30O2 |
| 8.94 | 786.6 | PC(18:1(9Z)/18:1(9Z)) | C44H84NO8P |
| 9.94 | 338.3406 | Erucamide | C22H43NO |
| 10.21 | 285.2774 | Stearic acid | C18H36O2 |
| 10.39 | 788.6126 | 1-Octadecanoyl-2-octadecenoyl-sn-glycero-3-phosphocholine | C44H86NO8P |
| 10.44 | 256.2626 | Palmitamide | C16H33NO |
| 10.6 | 282.278 | Oleamide | C18H35NO |
| 10.81 | 760.5849 | 2-Oleoyl-1-palmitoyl-sn-glycero-3-phosphocholine. | C42H82NO8P |
| 10.86 | 279.2334 | Linoleic Acid | C18H32O2 |
| 11.4 | 255.2334 | Palmitic acid | C16H32O2 |
| 11.65 | 325.1847 | Dodecylbenzenesulfonic acid | C18H30O3S |
| 12 | 835.5348 | 1-Hexadecanoyl-2-(9Z-octadecenoyl)-sn-glycero-3-phospho-(1'-myo-inositol). | C43H81O13P |

**Table S3.** Metabolite profiling of seed samples of four P. dactylifera varieties (AJS, ARS, SRS, DNS) using GC/MS data. Containing different amino acids, fatty acids, carbohydrates, polyphenols compounds.

| **RT (min)** | **Quant mass** | **Metabolite name** | **Formula** | **Ontology** |
| --- | --- | --- | --- | --- |
| 8.486 | 117.07 | Propylene glycol | C3H8O2 | 1,2-diols |
| 9.133 | 147.0691 | Lactic acid | C3H6O3 | Alpha hydroxy acids and derivatives |
| 9.511 | 147.0679 | Glycolic acid | C2H4O3 | Alpha hydroxy acids and derivatives |
| 10.11 | 116.0912 | L-Alanine | C3H7NO2 | Alanine and derivatives |
| 10.788 | 147.0672 | Oxalic acid | C2H2O4 | Dicarboxylic acids and derivatives |
| 11.787 | 130.1041 | N-Methyl-DL-Alanine | C4H9NO2 | Alanine and derivatives |
| 12.505 | 216.09 | Malonic acid | C3H4O4 | Dicarboxylic acids and derivatives |
| 12.703 | 147.0669 | 1-Aminocyclopropane-1-carboxylic acid | C4H7NO2 | Alpha amino acids |
| 12.817 | 144.1233 | L-Valine | C5H11NO2 | Valine and derivatives |
| 12.985 | 160.0754 | DL-Glyceraldehyde | C3H6O3 | Monosaccharides |
| 13.351 | 131.0829 | 3-Hydroxyisovaleric acid | C4H8O3 | Beta hydroxy acids and derivatives |
| 13.433 | 233.106 | 4-hydroxybutyric acid | C4H8O3 | Hydroxy fatty acids |
| 13.776 | 73.05 | 1,3-Dihydroxyacetone dimer | C6H12O6 | 1,4-dioxanes |
| 13.944 | 179.0516 | Benzoic acid | C7H6O2 | Benzoic acids |
| 13.968 | 174.1121 | Ethanolamine | C2H7NO | 1,2-aminoalcohols |
| 14.119 | 192.0896 | Glycerol | C3H8O3 | Sugar alcohols |
| 14.172 | 158.1394 | L-Leucine | C6H13NO2 | Leucine and derivatives |
| 14.569 | 341.0144 | Z artifact | C6H13NO2 | Medium-chain fatty acids |
| 14.658 | 158.14 | L-Isoleucine | C6H13NO2 | Isoleucine and derivatives |
| 14.834 | 142.1069 | L- (-)-Proline | C5H9NO2 | Proline and derivatives |
| 14.934 | 174.1143 | Glycine | C2H5NO2 | Alpha amino acids |
| 15.055 | 102.072 | Gamma-aminobutyric acid | C4H9NO2 | Gamma amino acids and derivatives |
| 15.125 | 180.0465 | Nicotinic acid | C6H5NO2 | Pyridinecarboxylic acids |
| 15.474 | 147.0657 | Glyceric acid | C3H6O4 | Sugar acids and derivatives |
| 15.875 | 241.0854 | Uracil | C4H4N2O2 | Pyrimidones |
| 16.152 | 204.1296 | L-Serine | C3H7NO3 | Serine and derivatives |
| 16.343 | 156.1217 | Pipecolic acid | C6H11NO2 | Alpha amino acids |
| 16.408 | 215.1491 | Nonanoric acid(9:0) | C9H18O2 | Medium-chain fatty acids |
| 16.656 | 141.0874 | beta-Cyano-L-alanine | C4H6N2O2 | Alpha amino acids |
| 16.68 | 73.06039 | L-Threonine | C4H9NO3 | L-alpha-amino acids |
| 17.2 | 255.0945 | Thymine | C5H6N2O2 | Hydroxypyrimidines |
| 17.37 | 261.0918 | Glutaric acid | C5H8O4 | Dicarboxylic acids and derivatives |
| 17.776 | 73.06 | 2-deoxytetronic acid | C4H8O4 | Beta hydroxy acids and derivatives |
| 17.932 | 216.1335 | Succinic acid | C4H6O4 | Dicarboxylic acids and derivatives |
| 18.083 | 218.15 | D-Homoserine | C4H9NO3 | L-alpha-amino acids |
| 18.55 | 247.1059 | Citramalic acid | C5H8O5 | Hydroxy fatty acids |
| 19.001 | 73.06519 | DL-Malic acid | C4H6O5 | Beta hydroxy acids and derivatives |
| 19.138 | 86.025 | D-Threitol | C4H10O4 | Sugar alcohols |
| 19.666 | 232.1293 | L-Aspartic acid | C4H7NO4 | Aspartic acid and derivatives |
| 19.735 | 230.1453 | trans-4-Hydroxy-L-proline | C5H9NO3 | Proline and derivatives |
| 19.93 | 156.0865 | L-5-Oxoproline | C5H7NO3 | Alpha amino acids and derivatives |
| 20.153 | 84.03941 | Glutamic Acid | C5H9NO4 | Glutamic acid and derivatives |
| 20.304 | 292.1262 | Threonic acid | C4H8O5 | Sugar acids and derivatives |
| 20.647 | 275.1437 | 2-Isopropylmalic acid | C7H12O5 | Hydroxy fatty acids |
| 20.811 | 129.0643 | 2-hydroxyglutaric acid | C5H8O5 | Short-chain hydroxy acids and derivatives |
| 21.536 | 142.1083 | Ornithine | C5H12N2O2 | L-alpha-amino acids |
| 21.898 | 218.1125 | L-(-)-Phenylalanine | C9H11NO2 | Phenylalanine and derivatives |
| 22.025 | 267.0792 | 4-Hydroxybenzoic acid | C7H6O3 | Hydroxybenzoic acid derivatives |
| 22.09 | 103.0508 | D-(+)-Xylose | C5H10O5 | Pentoses |
| 22.466 | 257.1929 | Lauric acid | C12H24O2 | Medium-chain fatty acids |
| 22.496 | 103.0533 | D-(-)-Ribose | C5H10O5 | Pentoses |
| 22.704 | 73.06039 | L-Asparagine | C4H8N2O3 | Asparagine and derivatives |
| 22.925 | 217.1143 | Pentitol | C5H12O5 | Sugar alcohols |
| 23.222 | 299.0797 | Xylitol | C5H12O5 | Sugar alcohols |
| 23.264 | 217.1135 | Arabitol | C5H12O5 | Sugar alcohols |
| 23.342 | 117.0679 | L-Rhamnose | C6H12O5 | Hexoses |
| 23.469 | 260.1504 | alpha-aminoadipic acid | C6H11NO4 | L-alpha-amino acids |
| 24.057 | 254.0891 | Orotic acid | C5H4N2O4 | Pyrimidinecarboxylic acids |
| 24.134 | 218.0878 | Glucose-1-phosphate | C6H13O9P | Monosaccharide phosphates |
| 24.143 | 229.1109 | cis-Aconitic acid | C6H6O6 | Tricarboxylic acids and derivatives |
| 24.21 | 357.1054 | Glycerol-alpha-phosphate | C3H9O6P | Glycerophosphates |
| 24.328 | 217.1213 | Pentonic acid | C5H10O6 | Sugar acids and derivatives |
| 24.347 | 73.06 | Ribitol | C5H12O5 | Sugar alcohols |
| 24.576 | 297.0915 | Vanillic acid | C8H8O4 | M-methoxybenzoic acids and derivatives |
| 24.609 | 156.0813 | L-Glutamine | C5H10N2O3 | Alpha amino acids |
| 25.018 | 204.09 | Shikimic acid | C7H10O5 | Shikimic acids and derivatves |
| 25.035 | 231.1075 | 3-deoxyhexitol | C6H14O5 | Hexoses |
| 25.222 | 273.1179 | Citric acid | C6H8O7 | Tricarboxylic acids and derivatives |
| 25.228 | 73.07 | DL-Isocitric acid | C6H8O7 | Tricarboxylic acids and derivatives |
| 25.407 | 193.0319 | 3,4-dihydroxybenzoic acid | C7H6O4 | Hydroxybenzoic acid derivatives |
| 25.459 | 256.1696 | L-(+)-Arginine | C6H14N4O2 | L-alpha-amino acids |
| 25.604 | 73.06 | D-Tagatose | C6H12O6 | Monosaccharides |
| 25.92 | 307.2 | Tagatose | C6H12O6 | Monosaccharides |
| 26.112 | 147.1089 | Psicose | C6H12O6 | Monosaccharides |
| 26.156 | 308.21 | D-(-)-Fructose | C6H12O6 | Monosaccharides |
| 26.159 | 308.21 | L-(-)-Sorbose | C6H12O6 | Monosaccharides |
| 26.409 | 147.12 | D-(+)-Allose | C6H12O6 | Hexoses |
| 26.413 | 117.0886 | D-Mannose | C6H12O6 | Hexoses |
| 26.416 | 205.178 | D-(+)-Galactose | C6H12O6 | Hexoses |
| 26.59 | 217.1164 | Gluconic acid lactone minor | C6H10O6 | Gluconolactones |
| 26.687 | 319.178 | Glucose | C6H12O6 | Hexoses |
| 26.915 | 205.1383 | D-(-)-Mannitol | C6H14O6 | Sugar alcohols |
| 27.005 | 174.115 | L-(+)-Lysine | C6H14N2O2 | D-alpha-amino acids |
| 27.081 | 362.15 | D-(+)-Galactosamine | C6H13NO5 | Hexoses |
| 27.148 | 204.1 | 1-methylgalactose | C7H14O6 | O-glycosyl compounds |
| 27.232 | 318.1355 | L-Iditol | C6H14O6 | Sugar alcohols |
| 27.343 | 203.1187 | D-(+)-Glucosamine | C6H13NO5 | Hexoses |
| 27.357 | 218.11 | Tyrosine | C9H11NO3 | Tyrosine and derivatives |
| 27.946 | 73.06 | Mannonic acid | C6H12O7 | Medium-chain hydroxy acids and derivatives |
| 28.183 | 292.1279 | Gluconic acid | C6H12O7 | Medium-chain hydroxy acids and derivatives |
| 28.208 | 291.1214 | Galactonic acid | C6H12O7 | Medium-chain hydroxy acids and derivatives |
| 28.281 | 297.1246 | 3-hydroxy-3-(4'-hydroxy-3'-methoxyphenyl)propionic acid | C19H36O5Si3 | Anisoles |
| 28.596 | 291.1439 | Glucosaminic acid | C6H13NO6 | D-alpha-amino acids |
| 28.67 | 516.2032 | Galacturonic acid | C6H10O7 | Glucuronic acid derivatives |
| 29.259 | 73.05 | Palmitic acid | C16H32O2 | Long-chain fatty acids |
| 29.417 | 73.06 | N-Acetyl-D-glucosamine | C8H15NO6 | N-acyl-alpha-hexosamines |
| 29.699 | 73.06 | Inositol | C6H12O6 | Cyclohexanols |
| 30.105 | 338.1181 | Ferulic acid | C10H10O4 | Hydroxycinnamic acids |
| 30.613 | 396.1438 | Caffeic acid | C9H8O4 | Hydroxycinnamic acids |
| 31.79 | 337.2372 | Linoleic acid | C18H32O2 | Lineolic acids and derivatives |
| 31.82 | 202.105 | L-Tryptophan | C11H11N2O2 | Indolyl carboxylic acids and derivatives |
| 31.863 | 339.2575 | Oleic acid | C18H34O2 | Long-chain fatty acids |
| 32.228 | 341.2819 | Stearic acid(17:0) | C18H36O2 | Long-chain fatty acids |
| 32.375 | 289.13 | Sinapic acid | C11H12O5 | Hydroxycinnamic acids |
| 32.688 | 204.1064 | Glycerol-3-galactoside | C9H18O8 | Glycosylglycerols |
| 32.778 | 387.1295 | D-Fructose 6-phosphate | C6H13O9P | Hexose phosphates |
| 33.732 | 217.1125 | Glucoheptulose | C7H14O7 | Heptoses |
| 35.102 | 174.1062 | Serotonin | C10H11N2O | NA |
| 36.425 | 361.1592 | Salicyl alcohol-b-glucoside | C13H18O7 | Phenolic glycosides |
| 37.095 | 204.1089 | Phytosphingosine | C18H39NO3 | 1,3-aminoalcohols |
| 37.388 | 236.103 | Adenosine | C10H13N5O4 | Purine nucleosides |
| 37.947 | 361.16 | alpha-Lactose | C12H22O11 | O-glycosyl compounds |
| 38.428 | 361.1613 | Sucrose | C12H22O11 | O-glycosyl compounds |
| 38.441 | 361.16 | D-(+)-Maltose | C12H22O11 | O-glycosyl compounds |
| 38.502 | 217.115 | Cellobiose | C12H22O11 | O-glycosyl compounds |
| 38.614 | 319.1476 | D-(+)-Trehalose | C12H22O11 | O-glycosyl compounds |
| 38.773 | 369.1385 | D-(+)-Turanose | C12H22O11 | O-glycosyl compounds |
| 38.811 | 204.1029 | Lactobionic acid | C12H22O12 | Fatty acyl glycosides of mono- and disaccharides |
| 39.101 | 204.1052 | Lactitol | C12H24O11 | Fatty acyl glycosides of mono- and disaccharides |
| 39.577 | 361.16 | Trehalose | C12H22O11 | O-glycosyl compounds |
| 39.762 | 361.1638 | Maltitol | C12H24O11 | Fatty acyl glycosides of mono- and disaccharides |
| 39.852 | 368.1593 | Epicatechin | C15H14O6 | Catechins |
| 40.115 | 368.16 | Catechin | C15H14O6 | Catechins |
| 40.135 | 204.1017 | Melibiose | C12H22O11 | O-glycosyl compounds |
| 40.236 | 204.1067 | Galactinol | C12H22O11 | O-glycosyl compounds |
| 45.2 | 361.165 | D-(+)-Raffinose | C18H32O16 | Oligosaccharides |
| 45.56 | 361.1635 | 1-kestose | C18H32O16 | Oligosaccharides |
| 45.926 | 361.1536 | Inulotriose | C18H32O16 | O-glycosyl compounds |
| 47.533 | 204.11 | Maltotriose | C18H32O16 | Oligosaccharides |
| 47.786 | 204.11 | D-Panose | C18H32O16 | Oligosaccharides |

**Table S4.** Metabolomic profiling of all four *P. dactylifera* cultivars of flesh samples of GC/MS data. Showing different classes of metabolites include amino acids, carbohydrates.

| **RT**  **(min)** | **Quant mass** | **Metabolite name** | **Formula** | **Ontology** |
| --- | --- | --- | --- | --- |
| 9.794 | 116.0847 | L-Alanine | C3H7NO2 | Alanine and derivatives |
| 10.522 | 147.0661 | Oxalic acid | C2H2O4 | Dicarboxylic acids and derivatives |
| 11.997 | 241.0428 | Methanolphosphate | CH5O4P | Monoalkyl phosphates |
| 12.592 | 57.07528 | 2-METHYL-1-HEXANOL | C7H16O | Fatty alcohols |
| 12.698 | 144.115 | L-Valine | C5H11NO2 | Valine and derivatives |
| 13.276 | 147.0667 | 4-hydroxybutyric acid | C4H8O3 | Hydroxy fatty acids |
| 13.889 | 174.1092 | Ethanolamine | C2H7NO | 1,2-aminoalcohols |
| 13.946 | 116.042 | L-Serine | C3H7NO3 | Serine and derivatives |
| 14.066 | 73.06 | Glycerol | C3H8O3 | Sugar alcohols |
| 14.53 | 341.0133 | Z artifact | C6H13NO2 | Medium-chain fatty acids |
| 14.776 | 142.104 | L-(-)-Proline | C5H9NO2 | Proline and derivatives |
| 14.879 | 174.1125 | Glycine | C2H5NO2 | Alpha amino acids |
| 15.324 | 147.0604 | Succinic acid | C4H6O4 | Dicarboxylic acids and derivatives |
| 15.438 | 73.05529 | Glyceric acid | C3H6O4 | Sugar acids and derivatives |
| 15.883 | 241.0638 | Uracil | C4H4N2O2 | Pyrimidones |
| 16.168 | 245.0722 | 3-Aminopropionitrile | C3H6N2 | Nitriles |
| 16.638 | 218.14 | L-Threonine | C4H9NO3 | L-alpha-amino acids |
| 18.614 | 218.1 | Aminomalonate | C3H5NO4 | Alpha amino acids |
| 18.984 | 73.05414 | L-(-)-Malic acid | C4H6O5 | Beta hydroxy acids and derivatives |
| 19.16 | 217.1067 | D-Threitol | C4H10O4 | Sugar alcohols |
| 19.652 | 232.125 | L-Aspartic acid | C4H7NO4 | Aspartic acid and derivatives |
| 19.845 | 174.11 | GABA | C4H9NO2 | Gamma amino acids and derivatives |
| 19.927 | 156.0824 | L-5-Oxoproline | C5H7NO3 | Alpha amino acids and derivatives |
| 20.29 | 292.125 | Isothreonic acid | C4H8O5 | Sugar acids and derivatives |
| 20.325 | 246.1327 | DL-threo-beta-Methylaspartic acid | C5H9NO4 | L-alpha-amino acids |
| 20.64 | 275.1335 | 2-Isopropylmalic acid | C7H12O5 | Hydroxy fatty acids |
| 20.788 | 129.063 | 2-hydroxyglutaric acid | C5H8O5 | Short-chain hydroxy acids and derivatives |
| 21.691 | 246.1362 | L-Glutamic acid | C5H9NO4 | Glutamic acid and derivatives |
| 22.013 | 204.1089 | Ribitol | C5H12O5 | Sugar alcohols |
| 22.026 | 200.1109 | Pipecolic acid | C6H11NO2 | Alpha amino acids |
| 22.21 | 103.0515 | D-(+)-Xylose | C5H10O5 | Pentoses |
| 22.256 | 207.1274 | METHYL 3,5-DICHLORO-2-HYDROXY-4-(2-PROPYNOXY)BENZOATE | C11H8Cl2O4 | o-Hydroxybenzoic acid esters |
| 22.491 | 73.05759 | D-(-)-Arabinose | C5H10O5 | Pentoses |
| 22.638 | 73.052 | L-Asparagine | C4H8N2O3 | Asparagine and derivatives |
| 23.226 | 217.116 | Xylitol | C5H12O5 | Sugar alcohols |
| 23.271 | 73.058 | Arabitol | C5H12O5 | Sugar alcohols |
| 23.346 | 117.0682 | L-Rhamnose | C6H12O5 | Hexoses |
| 23.437 | 117.0572 | D-(+)-Fucose | C6H12O5 | Hexoses |
| 23.687 | 174.106 | N-Acetylputrescine | C6H14N2O | Carboximidic acids |
| 23.999 | 231.12 | 3,6-anhydro-D-glucose minor | C6H10O5 | Tetrahydrofurans |
| 24.113 | 292.122 | Xylonic acid isomer | C5H10O6 | Sugar acids and derivatives |
| 24.154 | 217.0868 | Glucose-1-phosphate | C6H13O9P | Monosaccharide phosphates |
| 25.084 | 204.1037 | (-)-Shikimic acid | C7H10O5 | Shikimic acids and derivatves |
| 25.271 | 73.05 | DL-Isocitric acid | C6H8O7 | Tricarboxylic acids and derivatives |
| 25.581 | 307.14 | D-(-)-Fructose | C6H12O6 | Monosaccharides |
| 25.911 | 73.08 | S-Carboxymethyl-L-cysteine | C5H9NO4S | L-cysteine-S-conjugates |
| 25.927 | 307.2 | Tagatose | C6H12O6 | Monosaccharides |
| 25.969 | 308.2 | D-(-)-Fructose | C6H12O6 | Monosaccharides |
| 26.131 | 147.1022 | Psicose | C6H12O6 | Monosaccharides |
| 26.154 | 147.1087 | L-(-)-Sorbose | C6H12O6 | Monosaccharides |
| 26.426 | 319.2089 | D-Mannose | C6H12O6 | Hexoses |
| 26.442 | 205.18 | D-(+)-Galactose | C6H12O6 | Hexoses |
| 26.659 | 73.065 | L-Ascorbic acid | C6H8O6 | Butenolides |
| 26.681 | 319.1833 | Glucose | C6H12O6 | Hexoses |
| 26.985 | 73.05182 | L-Iditol | C6H14O6 | Sugar alcohols |
| 27.08 | 204.1025 | 1-methylgalactose | C7H14O6 | O-glycosyl compounds |
| 27.342 | 203.1158 | D-(+)-Glucosamine | C6H13NO5 | Hexoses |
| 27.963 | 333.1245 | Mannonic acid | C6H12O7 | Medium-chain hydroxy acids and derivatives |
| 28.2 | 292.0933 | Galactonic acid | C6H12O7 | Medium-chain hydroxy acids and derivatives |
| 28.249 | 305.132 | Myo-Inositol | C6H12O6 | Cyclohexanols |
| 29.312 | 313.2508 | Plamitic acid(16:0) | C16H32O2 | Long-chain fatty acids |
| 29.408 | 305.1369 | Inositol | C6H12O6 | Cyclohexanols |
| 29.938 | 174.1052 | Dopamine | C8H11NO2 | Catecholamines and derivatives |
| 30.043 | 319.1388 | N-acetylmannosamine | C8H15NO6 | N-acyl-alpha-hexosamines |
| 31.918 | 204.104 | beta-mannosylglycerate | C9H16O9 | Fatty acyl glycosides of mono- and disaccharides |
| 32.266 | 341.2808 | Stearic acid(17:0) | C18H36O2 | Long-chain fatty acids |
| 32.629 | 204.0931 | Glycerol-3-galactoside | C9H18O8 | Glycosylglycerols |
| 32.774 | 387.145 | D-Fructose 6-phosphate | C6H13O9P | Hexose phosphates |
| 33.628 | 73.051 | Glucoheptulose | C7H14O7 | Heptoses |
| 33.798 | 387.136 | Galactose-6-phosphate | C6H13O9P | Hexose phosphates |
| 35.078 | 174.0971 | Serotonin | C10H11N2O | NA |
| 36.244 | 361.1631 | alpha-Lactose | C12H22O11 | O-glycosyl compounds |
| 36.468 | 217.1167 | Salicyl alcohol-b-glucoside | C13H18O7 | Phenolic glycosides |
| 36.773 | 361.22 | D-(+)-Maltose | C12H22O11 | O-glycosyl compounds |
| 37.442 | 217.1158 | Sucrose | C12H22O11 | O-glycosyl compounds |
| 38.501 | 217.1157 | D-(+)-Trehalose | C12H22O11 | O-glycosyl compounds |
| 38.813 | 307.1419 | D-(+)-Turanose | C12H22O11 | O-glycosyl compounds |
| 39.571 | 361.1609 | Lactobionic acid | C12H22O12 | Fatty acyl glycosides of mono- and disaccharides |
| 39.941 | 361.1571 | Melibiose | C12H22O11 | O-glycosyl compounds |
| 40.065 | 204.1017 | Lactitol | C12H24O11 | Fatty acyl glycosides of mono- and disaccharides |
| 41.087 | 204.11 | Gentiobiose | C12H22O11 | O-glycosyl compounds |
| 42.558 | 204.1 | Galactinol | C12H22O11 | O-glycosyl compounds |
| 44.831 | 361.16 | Maltotriose | C18H32O16 | Oligosaccharides |
| 45.366 | 204.1078 | D-(+)-Raffinose | C18H32O16 | Oligosaccharides |
| 45.387 | 361.1583 | 1-Kestose | C19H34O16 | O-glycosyl compounds |
| 45.924 | 361.1641 | Inulotriose | C18H32O16 | O-glycosyl compounds |

**Table S5.** Significance analysis (p<0.05 and FDR) of date seed 40VIP scores (of all known and unknown compounds); the LC MS data.

| **Metabolite Name (40 VIP Scores)** | **Comp. 1** | **Comp. 2** | **Comp. 3** | **Comp. 4** | **Comp. 5** | **p.value** | **FDR** |
| --- | --- | --- | --- | --- | --- | --- | --- |
| 2-(2-hydroxyethoxy)ethanol;2-[2-(2-hydroxyethoxy)ethoxy]ethanol;2-methylprop-2-enoate | 3.4 | 3.2 | 3.2 | 3.2 | 3.2 | 1.92E-06 | 3.88E-05 |
| 4-methyleneglutamic acid | 3.4 | 3.1 | 3.1 | 3.1 | 3.1 | 6.18E-08 | 2.10E-06 |
| Umbelliferone | 3.3 | 3.1 | 3.0 | 3.0 | 3.0 | 3.88E-07 | 9.96E-06 |
| Caffeic Acid | 3.2 | 3.0 | 3.0 | 3.0 | 3.0 | 8.45E-05 | 0.000795 |
| Stigmastane steroids | 3.2 | 3.0 | 3.0 | 3.0 | 3.0 | 1.34E-07 | 4.00E-06 |
| 337.08798 Da 235.25 s | 3.1 | 2.9 | 2.8 | 2.8 | 2.8 | 0.000334 | 0.002472 |
| 5-O-Caffeoylshikimic acid | 3.1 | 2.9 | 2.8 | 2.8 | 2.8 | 0.00036 | 0.002624 |
| Tsangane L 3-glucoside | 3.0 | 2.8 | 2.8 | 2.8 | 2.8 | 8.14E-05 | 0.000777 |
| 866.20358 Da 202.88 s | 3.0 | 2.8 | 2.7 | 2.7 | 2.7 | 1.23E-05 | 0.000174 |
| Glutamic Acid | 2.9 | 2.7 | 2.7 | 2.7 | 2.7 | 6.26E-05 | 0.00064 |
| 290.94443 Da 124.00 s | 2.9 | 2.7 | 2.7 | 2.7 | 2.7 | 2.02E-05 | 0.000264 |
| Ginsenoside | 2.9 | 2.7 | 2.7 | 2.7 | 2.7 | 7.96E-10 | 7.59E-08 |
| 358.06672 Da 236.31 s | 2.9 | 2.7 | 2.6 | 2.6 | 2.6 | 0.001736 | 0.009883 |
| 487.18969 Da 46.01 s | 2.8 | 2.6 | 2.6 | 2.6 | 2.6 | 4.04E-08 | 1.56E-06 |
| 380.16860 Da 239.92 s | 2.8 | 2.6 | 2.6 | 2.6 | 2.6 | 3.23E-05 | 0.000388 |
| Quinazoline, 4-(2-quinolinylthio) | 2.8 | 2.6 | 2.6 | 2.6 | 2.6 | 0.000293 | 0.002236 |
| 186.02946 Da 137.56 s | 2.7 | 2.5 | 2.5 | 2.5 | 2.5 | 0.002925 | 0.015345 |
| Hovenitin I | 2.7 | 2.5 | 2.5 | 2.5 | 2.5 | 0.001722 | 0.009829 |
| Dihydrokaempferol | 2.7 | 2.5 | 2.5 | 2.5 | 2.5 | 4.01E-05 | 0.00046 |
| 769.17312 Da 224.37 s | 2.7 | 2.5 | 2.5 | 2.5 | 2.5 | 0.002593 | 0.014004 |
| 2-Oxoglutaric acid | 2.7 | 2.5 | 2.5 | 2.5 | 2.5 | 0.000873 | 0.005553 |
| L-Pyroglutamic acid | 2.7 | 2.5 | 2.5 | 2.5 | 2.5 | 0.0022 | 0.012146 |
| Syringic acid | 2.7 | 2.5 | 2.5 | 2.5 | 2.5 | 0.000517 | 0.00355 |
| Epiafzelechin 3-O-gallate | 2.6 | 2.5 | 2.4 | 2.4 | 2.4 | 0.000442 | 0.003119 |
| 474.17018 Da 39.26 s | 2.6 | 2.5 | 2.4 | 2.4 | 2.4 | 5.46E-05 | 0.000585 |
| 136.10007 Da 199.02 s | 2.6 | 2.5 | 2.4 | 2.4 | 2.4 | 1.92E-09 | 1.42E-07 |
| 259.10982 Da 43.30 s | 2.6 | 2.5 | 2.4 | 2.4 | 2.4 | 0.005101 | 0.023882 |
| Procyanidin B2 | 2.6 | 2.4 | 2.4 | 2.4 | 2.4 | 0.000535 | 0.00366 |
| Arginine | 2.6 | 2.4 | 2.4 | 2.4 | 2.4 | 3.21E-05 | 0.000388 |
| 117.93558 Da 191.13 s | 2.6 | 2.4 | 2.4 | 2.4 | 2.4 | 0.000332 | 0.002462 |
| 578.25750 Da 248.16 s | 2.6 | 2.4 | 2.5 | 2.4 | 2.4 | 1.37E-06 | 2.92E-05 |
| 145.95396 Da 823.98 s | 2.6 | 2.4 | 2.4 | 2.4 | 2.4 | 0.003149 | 0.016018 |
| 1793.38875 Da 219.11 s | 2.6 | 2.4 | 2.4 | 2.4 | 2.4 | 0.009125 | 0.039327 |
| 1444.33739 Da 221.73 s | 2.5 | 2.4 | 2.4 | 2.4 | 2.4 | 0.003346 | 0.016804 |
| 1730.39310 Da 221.17 s | 2.5 | 2.4 | 2.4 | 2.4 | 2.4 | 0.003615 | 0.017886 |
| 579.14523 Da 199.23 s | 2.5 | 2.4 | 2.3 | 2.3 | 2.3 | 0.000442 | 0.003119 |
| 961.54955 Da 232.72 s | 2.5 | 2.4 | 2.3 | 2.3 | 2.3 | 0.004352 | 0.020718 |
| 1505.32629 Da 211.85 s | 2.5 | 2.4 | 2.3 | 2.3 | 2.3 | 0.00112 | 0.006803 |
| 146.07637 Da 40.80 s | 2.5 | 2.3 | 2.3 | 2.3 | 2.3 | 3.58E-08 | 1.45E-06 |
| 864.85980 Da 227.29 s | 2.5 | 2.4 | 2.3 | 2.3 | 2.3 | 0.002825 | 0.015016 |

**Table S6.** Significance analysis (p<0.05 and FDR) of date flesh 40VIP scores (of all known and unknown compounds), the LC MS data.

| **Metabolite Name**  **(40 VIP Scores)** | **Comp. 1** | **Comp. 2** | **Comp. 3** | **Comp. 4** | **Comp. 5** | **p.value** | **FDR** |
| --- | --- | --- | --- | --- | --- | --- | --- |
| Aldehydo-D-glucose | 2.7 | 2.6 | 2.6 | 2.6 | 2.6 | 6.26E-12 | 8.61E-10 |
| D-Mannose 6-phosphate | 2.6 | 2.6 | 2.5 | 2.5 | 2.5 | 1.64E-11 | 1.78E-09 |
| 274.98516 Da 56.92 s | 2.6 | 2.5 | 2.5 | 2.5 | 2.5 | 1.58E-11 | 1.78E-09 |
| 440.09319 Da 84.93 s | 2.6 | 2.5 | 2.5 | 2.5 | 2.5 | 7.55E-12 | 9.74E-10 |
| Adenine | 2.6 | 2.5 | 2.5 | 2.5 | 2.5 | 7.39E-10 | 3.17E-08 |
| 456.10862 Da 76.74 s | 2.6 | 2.5 | 2.5 | 2.5 | 2.5 | 1.96E-09 | 7.19E-08 |
| 413.03843 Da 59.76 s | 2.5 | 2.5 | 2.5 | 2.5 | 2.5 | 8.58E-09 | 2.02E-07 |
| 298.08995 Da 55.54 s | 2.5 | 2.5 | 2.4 | 2.4 | 2.4 | 5.66E-11 | 4.67E-09 |
| 4.alpha.-Mannobiose | 2.5 | 2.5 | 2.4 | 2.4 | 2.4 | 1.08E-09 | 4.38E-08 |
| Xylosyl-cellobiose | 2.5 | 2.5 | 2.4 | 2.4 | 2.4 | 4.40E-13 | 1.37E-10 |
| Arginine | 2.5 | 2.4 | 2.4 | 2.4 | 2.4 | 1.19E-10 | 8.21E-09 |
| Diphenylsulfoxide | 2.5 | 2.5 | 2.4 | 2.4 | 2.4 | 5.37E-12 | 7.91E-10 |
| 1-[(Dimethoxymethoxy-hydroxy-methoxymethoxy)-hydroxy-methoxymethoxy]-1-methoxyethanol | 2.5 | 2.4 | 2.4 | 2.4 | 2.4 | 3.64E-09 | 1.14E-07 |
| 328.01710 Da 94.24 s | 2.5 | 2.4 | 2.4 | 2.4 | 2.4 | 5.38E-10 | 2.41E-08 |
| 405.11211 Da 141.34 s | 2.5 | 2.4 | 2.4 | 2.4 | 2.4 | 5.18E-08 | 9.89E-07 |
| Diethyl formamidomalonate | 2.5 | 2.4 | 2.4 | 2.4 | 2.4 | 2.80E-10 | 1.48E-08 |
| Erythronic acid | 2.5 | 2.4 | 2.4 | 2.4 | 2.4 | 6.49E-09 | 1.65E-07 |
| 548.06345 Da 62.07 s | 2.5 | 2.4 | 2.4 | 2.4 | 2.4 | 4.83E-09 | 1.32E-07 |
| 307.21030 Da 29.75 s | 2.5 | 2.4 | 2.4 | 2.4 | 2.4 | 1.06E-07 | 1.78E-06 |
| 1-tert-butylsulfanylsulfonyl-4-methylbenzene | 2.5 | 2.4 | 2.4 | 2.4 | 2.4 | 2.18E-08 | 4.45E-07 |
| N-(4-Aminobutyl)-1-[(2R,5R)-2-(phosphonooxymethyl)-1,3-oxathiolane-5-yl]cytosine | 2.4 | 2.4 | 2.3 | 2.3 | 2.3 | 1.40E-07 | 2.24E-06 |
| 481.18949 Da 46.57 s | 2.4 | 2.4 | 2.3 | 2.3 | 2.3 | 5.75E-08 | 1.08E-06 |
| 522.17987 Da 48.61 s | 2.4 | 2.4 | 2.3 | 2.3 | 2.3 | 9.62E-07 | 1.08E-05 |
| 342.11730 Da 47.92 s | 2.4 | 2.4 | 2.3 | 2.3 | 2.3 | 1.65E-08 | 3.50E-07 |
| Bis(1-piperidinecarbodithioic acid)2-hydroxypropane-1,3-diyl ester | 2.4 | 2.3 | 2.3 | 2.3 | 2.3 | 2.40E-07 | 3.48E-06 |
| 4-(purin-6-ylamino)benzoic acid | 2.4 | 2.3 | 2.3 | 2.3 | 2.3 | 4.66E-07 | 5.86E-06 |
| (2R)-3-Hydroxy-2-(?-D-mannopyranosyloxy)propanoic acid | 2.4 | 2.3 | 2.3 | 2.3 | 2.3 | 1.61E-07 | 2.49E-06 |
| 159.08920 Da 43.59 s | 2.4 | 2.3 | 2.3 | 2.3 | 2.3 | 2.85E-09 | 9.48E-08 |
| Galactinol | 2.4 | 2.3 | 2.3 | 2.3 | 2.3 | 2.05E-14 | 1.94E-11 |
| 498.12010 Da 64.21 s | 2.4 | 2.3 | 2.3 | 2.3 | 2.3 | 5.61E-08 | 1.06E-06 |
| 414.04339 Da 51.76 s | 2.4 | 2.3 | 2.3 | 2.3 | 2.3 | 4.01E-06 | 3.71E-05 |
| 278.04068 Da 84.61 s | 2.4 | 2.3 | 2.3 | 2.3 | 2.3 | 6.53E-07 | 7.78E-06 |
| 430.13213 Da 142.50 s | 2.4 | 2.3 | 2.3 | 2.3 | 2.3 | 2.01E-11 | 2.07E-09 |
| 469.17805 Da 51.57 s | 2.4 | 2.3 | 2.3 | 2.3 | 2.3 | 1.34E-07 | 2.15E-06 |
| 458.12687 Da 135.71 s | 2.4 | 2.3 | 2.3 | 2.3 | 2.3 | 4.86E-09 | 1.32E-07 |
| 351.15187 Da 49.29 s | 2.3 | 2.3 | 2.3 | 2.3 | 2.3 | 2.41E-11 | 2.36E-09 |
| 83.03686 Da 39.63 s | 2.3 | 2.3 | 2.3 | 2.3 | 2.3 | 1.48E-05 | 0.000115 |
| 277.11638 Da 49.24 s | 2.3 | 2.3 | 2.3 | 2.3 | 2.3 | 3.66E-07 | 4.97E-06 |
| 282.01079 Da 97.81 s | 2.3 | 2.3 | 2.3 | 2.3 | 2.3 | 1.89E-08 | 3.95E-07 |
| 440.08362 Da 284.90 s | 2.3 | 2.3 | 2.3 | 2.2 | 2.2 | 6.90E-07 | 8.14E-06 |

**Table S7.** Significance analysis (p<0.05 and FDR) of date seed 40VIP Scores (of all known compounds only), the LC MS data.

| **Metabolite Name (40 VIP Scores)** | **Comp. 1** | **Comp. 2** | **Comp. 3** | **Comp. 4** | **Comp. 5** | **Comp. 6** | **Comp. 7** | **Comp. 8** | **p.value** | **FDR** |
| --- | --- | --- | --- | --- | --- | --- | --- | --- | --- | --- |
| Umbelliferone | 2.5 | 2.4 | 2.4 | 2.3 | 2.3 | 2.3 | 2.3 | 2.3 | 0.002159 | 0.00474 |
| Caffeic Acid | 2.3 | 2.2 | 2.2 | 2.2 | 2.2 | 2.2 | 2.2 | 2.2 | 0.002336 | 0.004937 |
| Arginine | 2.1 | 2.0 | 2.0 | 2.0 | 2.0 | 2.0 | 2.0 | 2.0 | 0.000185 | 0.000531 |
| Tsangane L 3-glucoside | 2.0 | 1.9 | 1.9 | 1.9 | 1.9 | 1.9 | 1.9 | 1.9 | 0.000251 | 0.000686 |
| Syringic acid | 1.9 | 1.8 | 1.8 | 1.8 | 1.8 | 1.8 | 1.8 | 1.8 | 0.000332 | 0.000843 |
| Dihydrokaempferol | 1.9 | 1.8 | 1.8 | 1.8 | 1.8 | 1.8 | 1.8 | 1.8 | 6.26E-05 | 0.0002 |
| DL-a-glycerophosphate | 1.8 | 1.7 | 1.7 | 1.7 | 1.7 | 1.7 | 1.7 | 1.7 | 0.010384 | 0.017621 |
| Palmitic Acid ethyl ester | 1.8 | 1.7 | 1.7 | 1.7 | 1.7 | 1.7 | 1.7 | 1.7 | 1.34E-05 | 5.17E-05 |
| (-)-Epicatechin | 1.8 | 1.7 | 1.7 | 1.7 | 1.7 | 1.7 | 1.7 | 1.7 | 0.000298 | 0.000777 |
| LPI 16:0 | 1.7 | 1.7 | 1.7 | 1.7 | 1.7 | 1.7 | 1.7 | 1.7 | 4.91E-06 | 2.39E-05 |
| NCGC00016415-07!(2R,3R)-2-(3,4-dihydroxyphenyl)-3,4-dihydro-2H-chromene-3,5,7-triol | 1.7 | 1.6 | 1.6 | 1.6 | 1.6 | 1.6 | 1.6 | 1.6 | 1.99E-06 | 1.21E-05 |
| Benzoic acid | 1.6 | 1.6 | 1.6 | 1.6 | 1.6 | 1.6 | 1.6 | 1.6 | 0.00099 | 0.002263 |
| Procyanidin C1 | 1.6 | 1.6 | 1.6 | 1.6 | 1.6 | 1.6 | 1.6 | 1.6 | 0.003734 | 0.006785 |
| 4-Decylbenzenesulfonic acid | 1.5 | 1.6 | 1.6 | 1.6 | 1.6 | 1.6 | 1.6 | 1.6 | 0.002471 | 0.005031 |
| Vanillic acid | 1.5 | 1.5 | 1.5 | 1.5 | 1.5 | 1.5 | 1.5 | 1.5 | 8.17E-07 | 6.10E-06 |
| Decaffeoyl-acteoside | 1.5 | 1.4 | 1.4 | 1.4 | 1.4 | 1.4 | 1.4 | 1.4 | 2.53E-09 | 4.59E-08 |
| Procyanidin B1 | 1.5 | 1.5 | 1.5 | 1.5 | 1.5 | 1.5 | 1.5 | 1.5 | 0.003756 | 0.006785 |
| Homogentisic acid | 1.5 | 1.4 | 1.4 | 1.4 | 1.4 | 1.4 | 1.4 | 1.4 | 8.50E-10 | 1.90E-08 |
| L-Aspartic Acid | 1.4 | 1.4 | 1.4 | 1.4 | 1.4 | 1.4 | 1.4 | 1.4 | 0.02477 | 0.038531 |
| 4-(Hexopyranosyloxy)-3-methoxybenzoic acid | 1.4 | 1.4 | 1.4 | 1.4 | 1.4 | 1.4 | 1.4 | 1.4 | 8.93E-05 | 0.000263 |
| 3,4-Dihydroxybenzaldehyde | 1.3 | 1.3 | 1.3 | 1.3 | 1.3 | 1.3 | 1.3 | 1.3 | 0.000419 | 0.00102 |
| 4-Hydroxybenzoic acid | 1.3 | 1.2 | 1.2 | 1.2 | 1.2 | 1.2 | 1.2 | 1.2 | 7.67E-08 | 7.81E-07 |
| CATECHOL | 1.2 | 1.2 | 1.2 | 1.2 | 1.2 | 1.2 | 1.2 | 1.2 | 1.87E-06 | 1.21E-05 |
| DL-Norleucine | 1.2 | 1.1 | 1.1 | 1.1 | 1.1 | 1.1 | 1.1 | 1.1 | 0.016163 | 0.025861 |
| Blumenin | 1.2 | 1.2 | 1.1 | 1.1 | 1.1 | 1.1 | 1.1 | 1.1 | 0.002226 | 0.004793 |
| Tyrosine | 1.2 | 1.1 | 1.1 | 1.1 | 1.1 | 1.1 | 1.1 | 1.1 | 0.022447 | 0.035409 |
| Citric acid | 1.1 | 1.0 | 1.0 | 1.0 | 1.0 | 1.0 | 1.0 | 1.0 | 0.003232 | 0.006223 |
| Erucamide | 1.1 | 1.0 | 1.0 | 1.0 | 1.0 | 1.0 | 1.0 | 1.0 | 0.010274 | 0.017621 |
| Threonic acid | 1.0 | 1.0 | 1.0 | 1.0 | 1.0 | 1.0 | 1.0 | 1.0 | 1.11E-05 | 4.61E-05 |
| L-Tyrosine | 1.0 | 1.0 | 1.0 | 1.0 | 1.0 | 1.0 | 1.0 | 1.0 | 3.81E-06 | 1.94E-05 |
| D-Galactose | 1.0 | 1.0 | 1.0 | 1.0 | 1.0 | 1.0 | 1.0 | 1.0 | 0.015421 | 0.025031 |
| PC 15:0_15:1 | 1.0 | 1.0 | 1.0 | 1.0 | 1.0 | 1.0 | 1.0 | 1.0 | 8.77E-05 | 0.000263 |
| Salicylic acid glucoside | 1.0 | 1.0 | 1.0 | 1.0 | 1.0 | 1.0 | 1.0 | 1.0 | 1.21E-07 | 1.13E-06 |

**Table S8.** Significance analysis (p<0.05 and FDR) of date flesh 40VIP Scores (of all known compounds only), the LC MS data.

| **Metabolite Name**  **(40 VIP Scores)** | **Comp1** | **Comp2** | **Comp3** | **Comp4** | **Comp5** | **Comp6** | **Comp7** | **Comp8** | **p.value** | **FDR** |
| --- | --- | --- | --- | --- | --- | --- | --- | --- | --- | --- |
|  |  |  |  |  |  |  |  |  |  |  |
| D-Mannose 6-phosphate | 1.9 | 1.8 | 1.8 | 1.8 | 1.8 | 1.8 | 1.8 | 1.8 | 1.58E-12 | 7.41E-11 |
| Arginine | 1.8 | 1.7 | 1.7 | 1.7 | 1.7 | 1.7 | 1.7 | 1.7 | 5.41E-11 | 8.48E-10 |
| Diphenylsulfoxide | 1.8 | 1.8 | 1.7 | 1.7 | 1.7 | 1.7 | 1.7 | 1.7 | 5.57E-12 | 1.05E-10 |
| Adenine | 1.8 | 1.7 | 1.7 | 1.7 | 1.7 | 1.7 | 1.7 | 1.7 | 2.74E-09 | 2.57E-08 |
| Threonic acid | 1.7 | 1.7 | 1.7 | 1.7 | 1.7 | 1.7 | 1.7 | 1.7 | 6.87E-10 | 8.07E-09 |
| Galactinol | 1.7 | 1.6 | 1.6 | 1.6 | 1.6 | 1.6 | 1.6 | 1.6 | 1.39E-13 | 1.31E-11 |
| 414.04339 Da 51.76 s | 1.7 | 1.6 | 1.6 | 1.6 | 1.6 | 1.6 | 1.6 | 1.6 | 2.88E-06 | 1.04E-05 |
| D-Arabinonic acid | 1.7 | 1.6 | 1.6 | 1.6 | 1.6 | 1.6 | 1.6 | 1.6 | 4.91E-06 | 1.65E-05 |
| Trehalose 6-phosphate | 1.7 | 1.6 | 1.6 | 1.6 | 1.6 | 1.6 | 1.6 | 1.6 | 2.48E-10 | 3.33E-09 |
| 277.11638 Da 49.24 s | 1.7 | 1.6 | 1.6 | 1.6 | 1.6 | 1.6 | 1.6 | 1.6 | 2.29E-07 | 1.20E-06 |
| alpha.'-Trehalose 6-phosphate | 1.7 | 1.6 | 1.6 | 1.6 | 1.6 | 1.6 | 1.6 | 1.6 | 2.45E-12 | 7.67E-11 |
| Sucrose | 1.6 | 1.5 | 1.5 | 1.5 | 1.5 | 1.5 | 1.5 | 1.5 | 9.90E-07 | 4.23E-06 |
| Adenosine | 1.6 | 1.5 | 1.5 | 1.5 | 1.5 | 1.5 | 1.5 | 1.5 | 3.20E-08 | 2.15E-07 |
| L-(-)-Phenylalanine | 1.5 | 1.4 | 1.4 | 1.4 | 1.4 | 1.4 | 1.4 | 1.4 | 6.08E-06 | 1.97E-05 |
| N2-Acetyl-L-aminoadipate | 1.5 | 1.5 | 1.5 | 1.5 | 1.5 | 1.5 | 1.5 | 1.5 | 0.000209 | 0.000416 |
| N-Fructosyl tyrosine | 1.5 | 1.4 | 1.4 | 1.4 | 1.4 | 1.4 | 1.4 | 1.4 | 1.07E-05 | 3.04E-05 |
| Pyrophosphate | 1.4 | 1.5 | 1.5 | 1.4 | 1.4 | 1.4 | 1.4 | 1.4 | 1.26E-05 | 3.49E-05 |
| Octanoic acid, 2-[[4-chloro-6-[(2,3-dimethylphenyl)amino]-2-pyrimidinyl]thio]- | 1.4 | 1.4 | 1.4 | 1.4 | 1.3 | 1.3 | 1.3 | 1.3 | 4.54E-05 | 0.000107 |
| Gamma-Aminobutyric acid | 1.4 | 1.4 | 1.4 | 1.4 | 1.4 | 1.4 | 1.4 | 1.4 | 9.47E-05 | 0.000207 |
| Ortophosphate | 1.4 | 1.4 | 1.4 | 1.4 | 1.4 | 1.4 | 1.4 | 1.4 | 2.37E-05 | 5.72E-05 |
| Serotonin | 1.4 | 1.4 | 1.4 | 1.4 | 1.4 | 1.4 | 1.4 | 1.4 | 6.21E-07 | 2.92E-06 |
| L-trans-alpha-Amino-2-carboxycyclopropaneacetic acid | 1.4 | 1.4 | 1.4 | 1.4 | 1.4 | 1.4 | 1.4 | 1.4 | 1.44E-05 | 3.76E-05 |
| DL-Phenylalanine | 1.4 | 1.4 | 1.4 | 1.3 | 1.3 | 1.3 | 1.3 | 1.3 | 1.47E-06 | 5.74E-06 |
| Fructose-L-tryptophan | 1.4 | 1.3 | 1.3 | 1.3 | 1.3 | 1.3 | 1.3 | 1.3 | 1.76E-06 | 6.60E-06 |
| D-(+)-Trehalose | 1.3 | 1.3 | 1.3 | 1.3 | 1.3 | 1.3 | 1.3 | 1.3 | 0.000105 | 0.000219 |
| N-Acryloyl-DL-aspartic acid | 1.3 | 1.3 | 1.3 | 1.3 | 1.3 | 1.3 | 1.3 | 1.3 | 1.44E-07 | 7.94E-07 |
| N-Fructosyl isoleucine | 1.3 | 1.3 | 1.3 | 1.3 | 1.3 | 1.3 | 1.3 | 1.3 | 3.90E-09 | 3.33E-08 |
| GLUCONATE | 1.3 | 1.2 | 1.2 | 1.2 | 1.2 | 1.2 | 1.2 | 1.2 | 1.10E-06 | 4.48E-06 |
| trifluoroacetic acid | 1.2 | 1.2 | 1.2 | 1.2 | 1.2 | 1.2 | 1.2 | 1.2 | 0.000596 | 0.001099 |
| Caffeic Acid | 1.2 | 1.2 | 1.2 | 1.2 | 1.2 | 1.2 | 1.2 | 1.2 | 1.36E-05 | 3.66E-05 |
| N-Acetylneuraminic Acid | 1.2 | 1.2 | 1.2 | 1.2 | 1.2 | 1.2 | 1.2 | 1.2 | 3.96E-12 | 9.30E-11 |
| N-Fructosyl phenylalanine | 1.2 | 1.2 | 1.2 | 1.2 | 1.2 | 1.2 | 1.2 | 1.2 | 5.55E-09 | 4.35E-08 |
| tianshic acid | 1.1 | 1 | 1 | 1 | 1 | 1 | 1 | 1 | 0.009564 | 0.014047 |
| L-Asparagine | 1.1 | 1.2 | 1.2 | 1.2 | 1.2 | 1.2 | 1.2 | 1.2 | 1.00E-05 | 2.95E-05 |
| DL-Phenylalanine' | 1 | 1 | 1 | 1 | 1 | 1 | 1 | 1 | 0.02676 | 0.036991 |
| 2-Ethylhexyl sebacate | 1 | 1 | 1 | 1 | 1 | 1 | 1 | 1 | 7.61E-09 | 5.50E-08 |
| Ethyl (S)-3-hydroxybutyrate glucoside | 1 | 1.1 | 1.1 | 1.1 | 1.1 | 1.1 | 1.1 | 1.1 | 7.61E-09 | 5.50E-08 |
| Pyrogallol | 1 | 1 | 1 | 1 | 1 | 1 | 1 | 1 | 0.002636 | 0.004505 |
| DL-threo-beta-Methylaspartic acid | 1 | 1.1 | 1.1 | 1.1 | 1.1 | 1.1 | 1.1 | 1.1 | 5.71E-07 | 2.83E-06 |
| Ferulic acid | 1 | 1 | 1 | 1 | 1 | 1 | 1 | 1 | 0.003296 | 0.005435 |


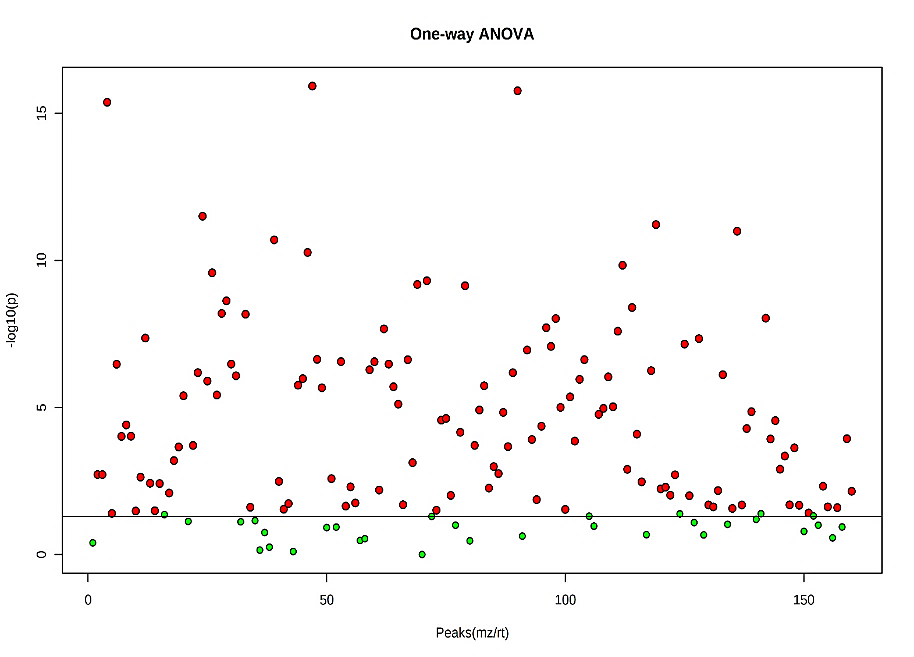

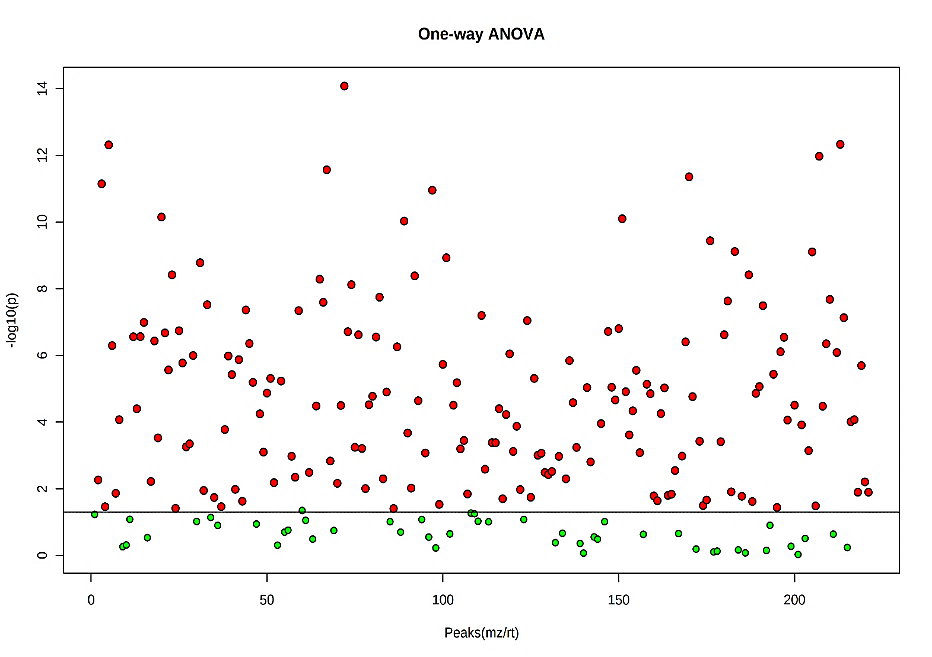

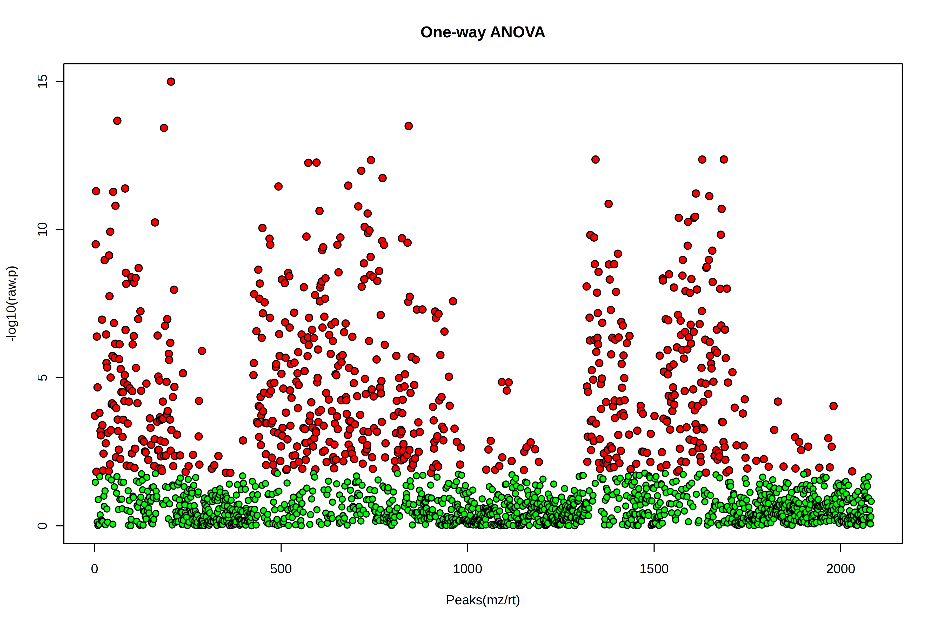

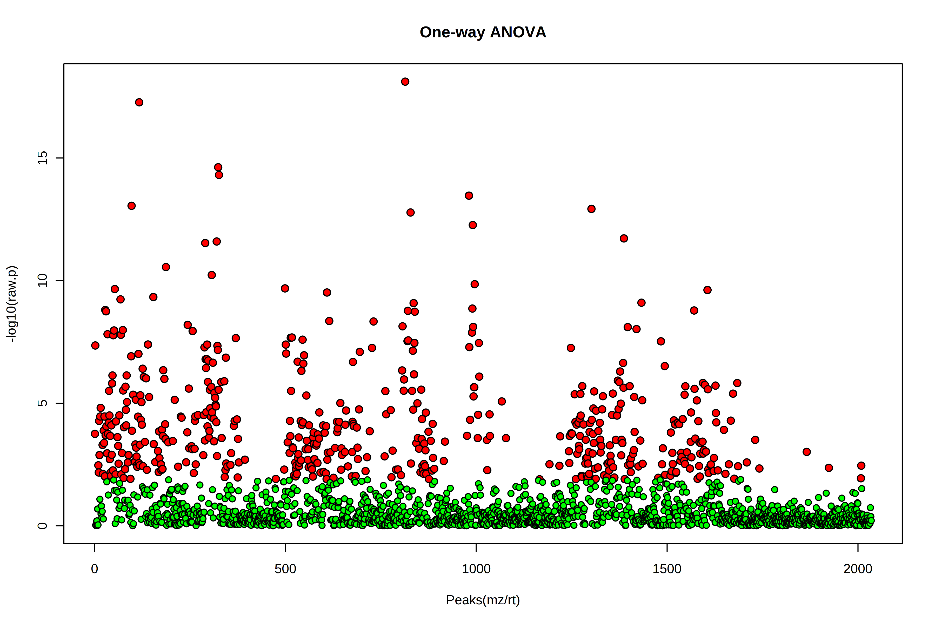


b

b

a

a

**Figure S2.** One-way ANOVA test of GC-MS data, illustrating the a) 173 significant metabolites (red circles) identified in seed samples, and b) 128 significant met in flesh samples

**Figure S1.** One-way ANOVA test of UHPLC-MS data, illustrating the a) 525 significant metabolites (red circles) identified in seed samples, and b) 686 significant molecules in the flesh samples.


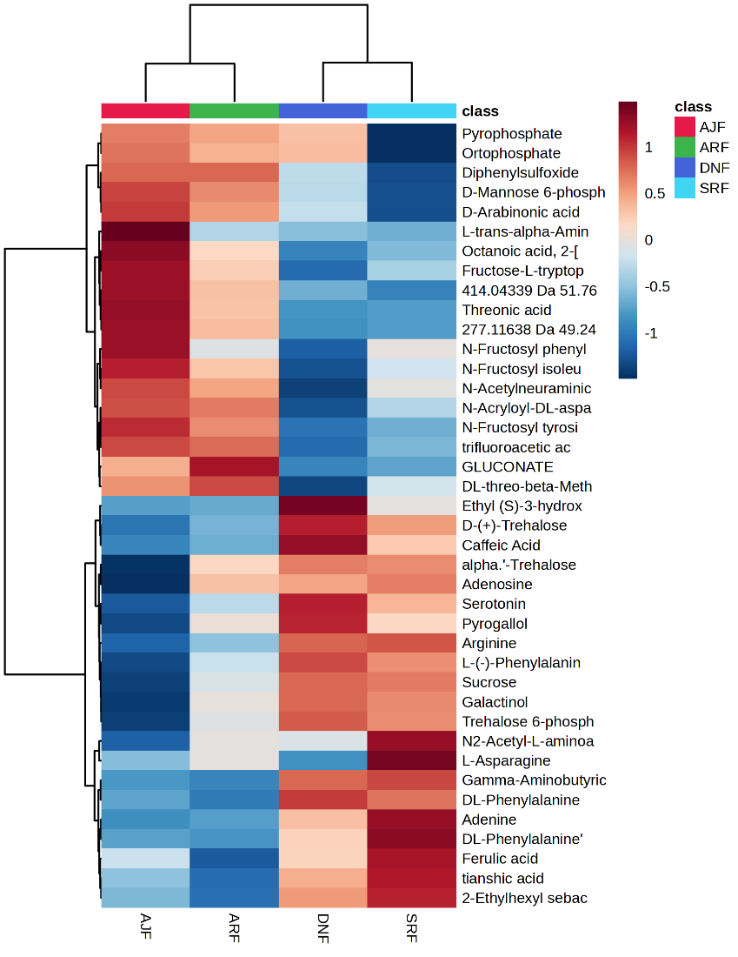

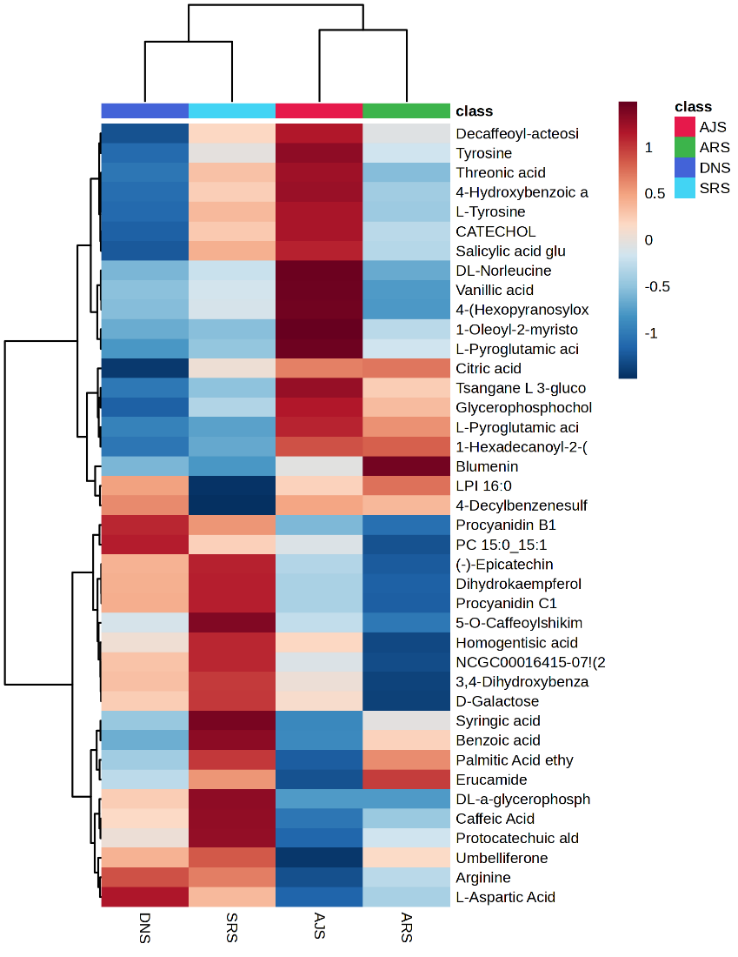


**Figure S3.** Heatmap visualization of 40 VIP-annotated compounds; (a) Heatmap representation of 40 VIP-annotated compounds from the flesh profiling analysis and (b) from the seed analysis.

a

b


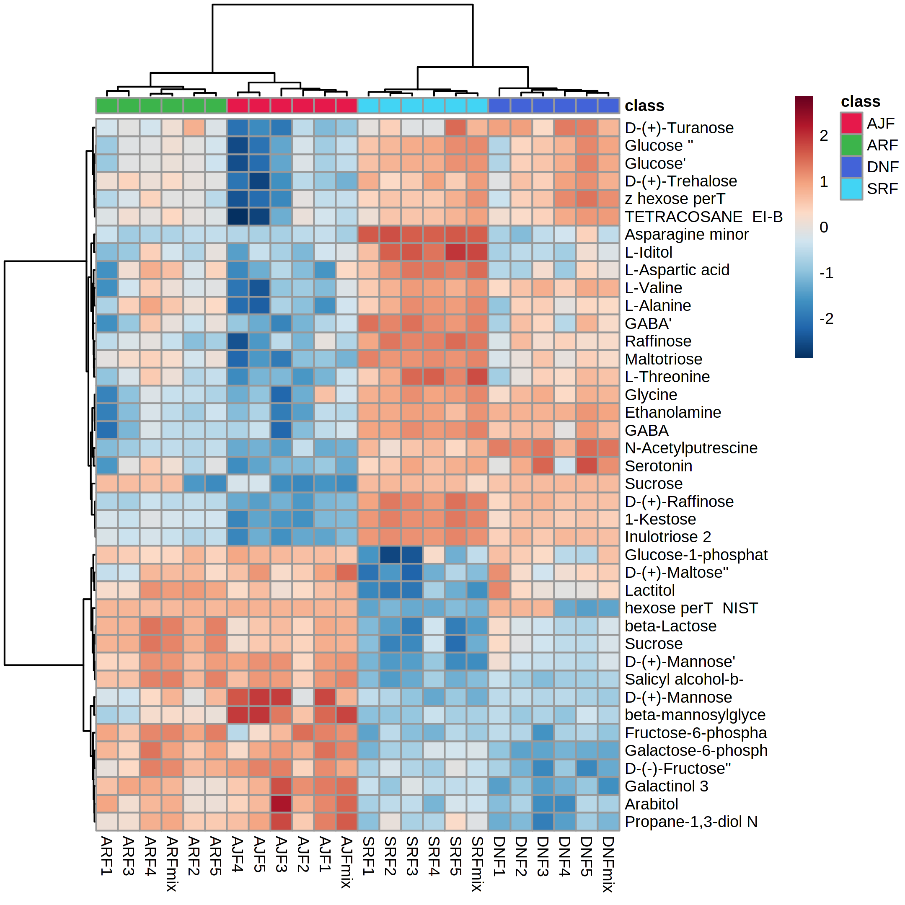

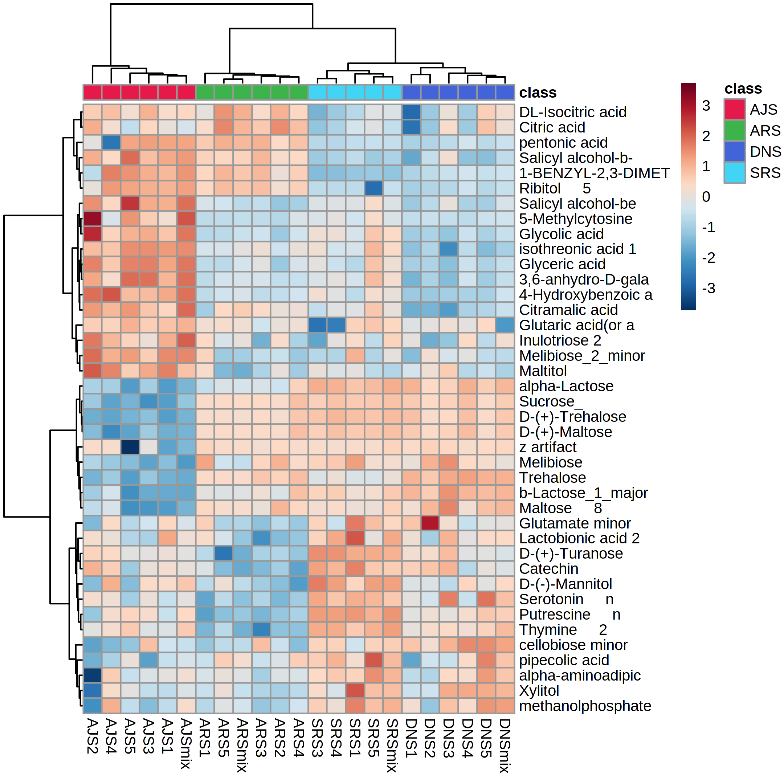

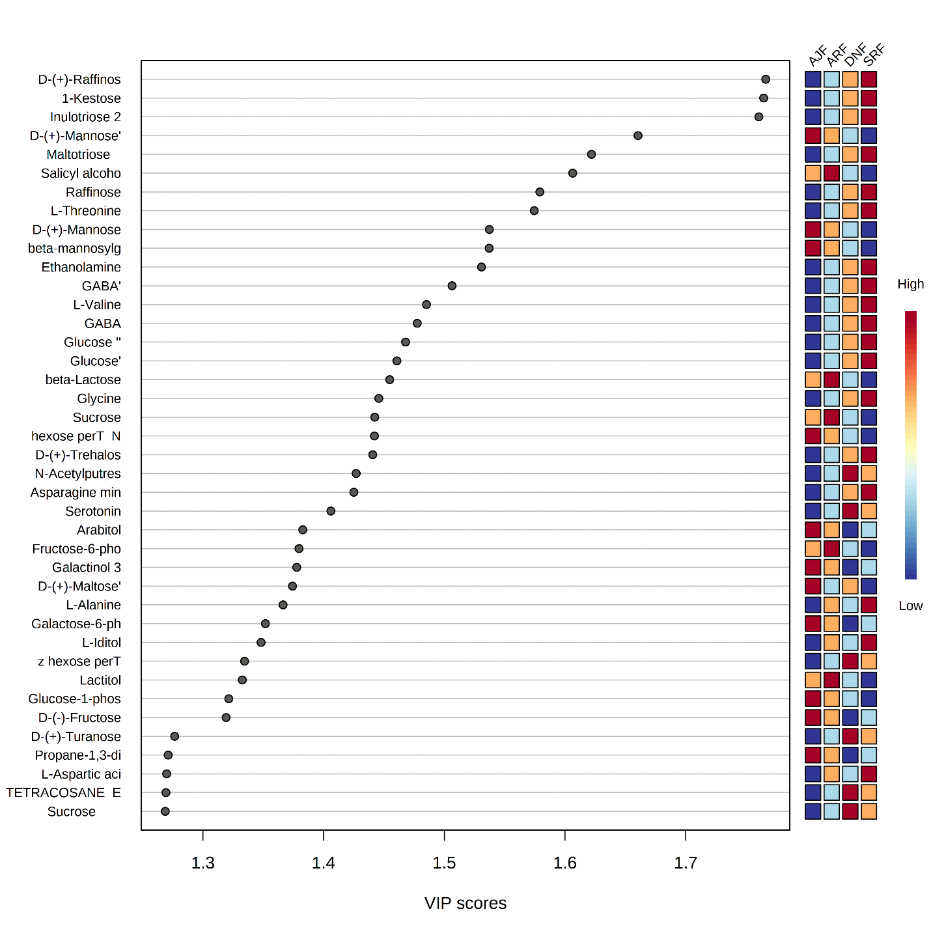

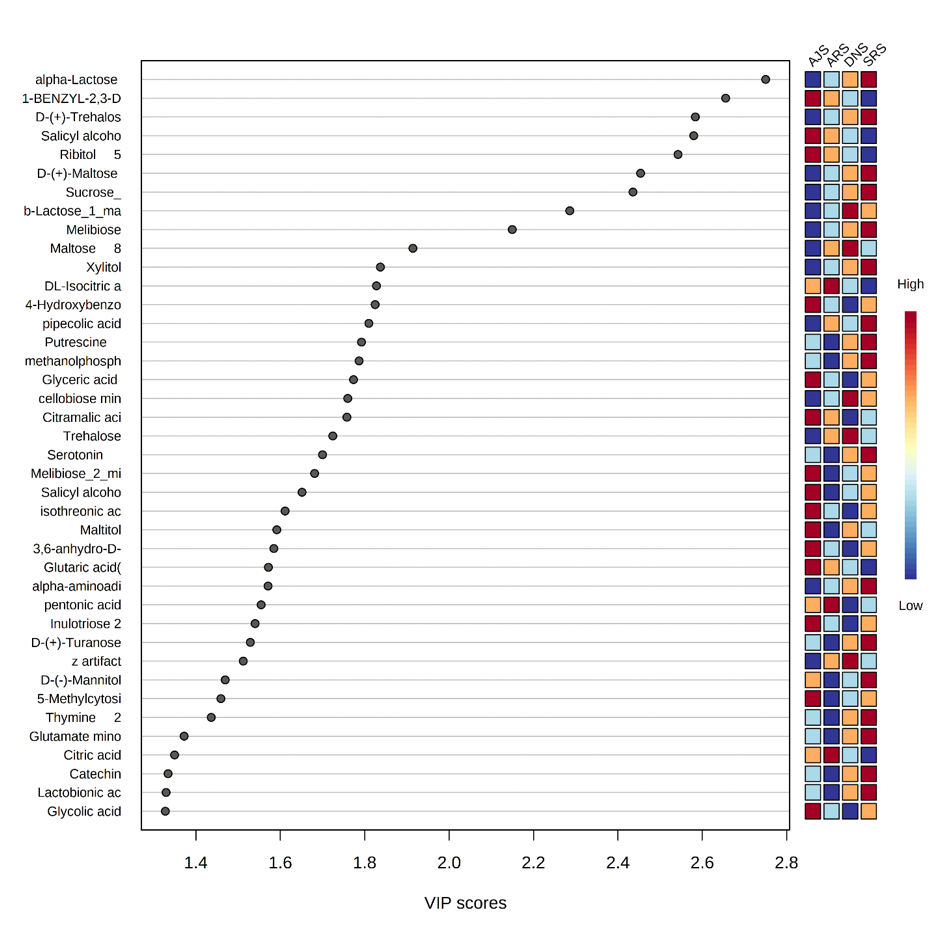


**Figure S4.** 40 VIP metabolites that significantly associate with date varieties segregation (GC-MS). a. seed, b. flesh.

a

b

**Figure S5.** HCA of the four date cultivars based on GC-MS data using MetaboAnalyst software. The heatmap shows date varieties a) seed samples, and b) flesh samples.

a

b


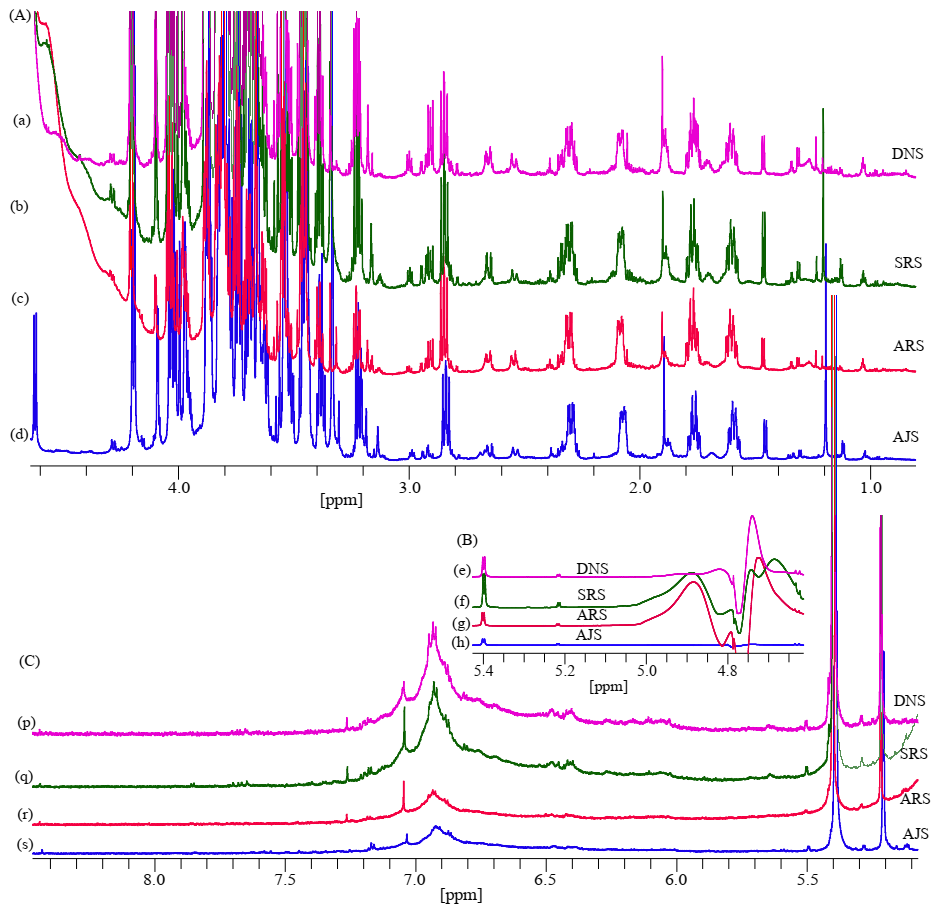


**Figure S6.** (A), (B), and (C) Display toggled expanded 1D 1H NMR spectra from the regions 0.78 to 4.61 ppm, 4.65 to 5.42 ppm, and 5.09 to 8.48 ppm, respectively of different palm date seeds as (a, e, p) for DNS, (b, f, q) for SRS, (c, g, r) for ARS, and (d, h, s) for AJS recorded by using solution-state NMR of 800 MHz in D2O solution.


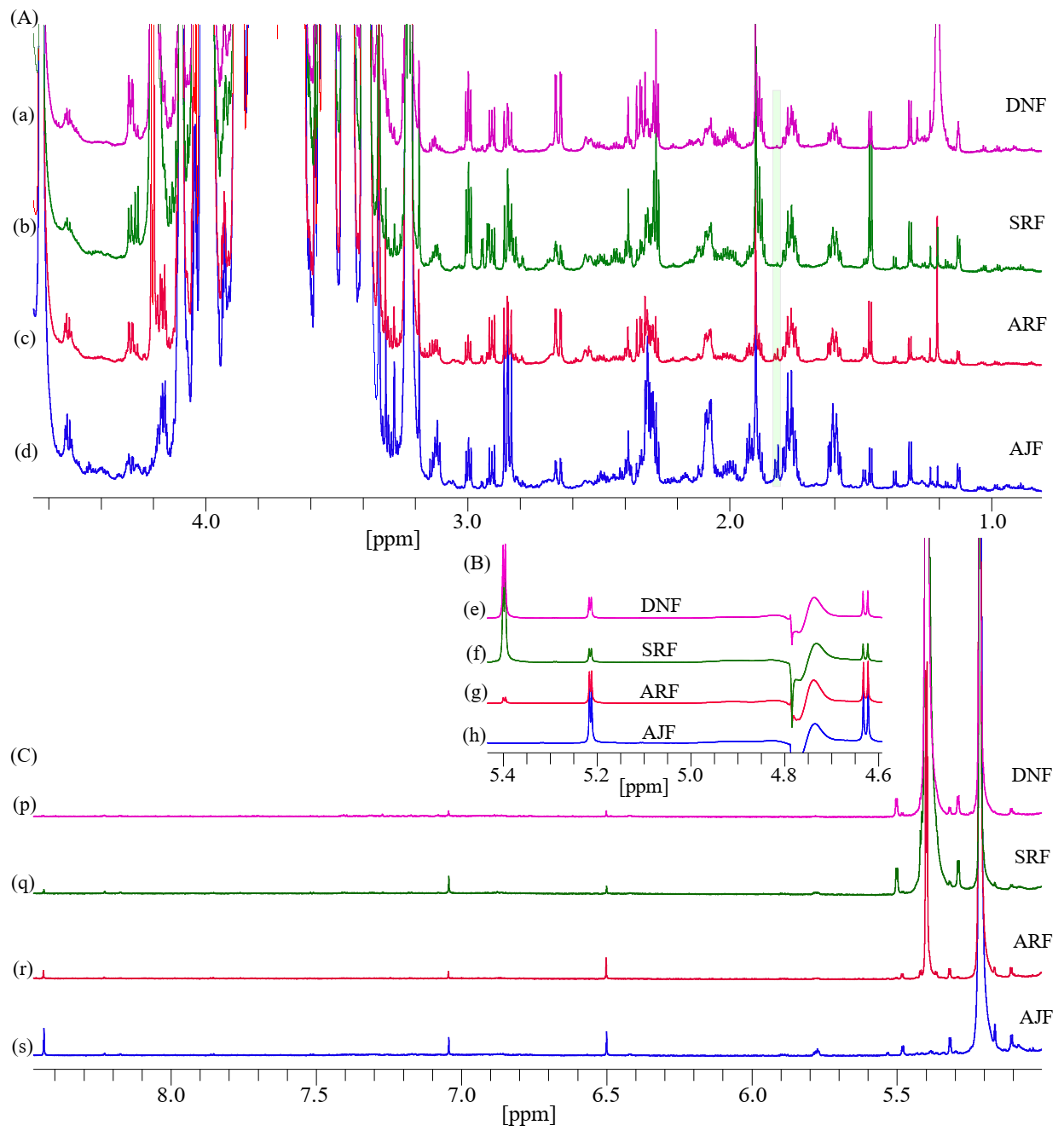


**Figure S7.** (A), (B), and (C) Display toggled expanded 1D 1H NMR spectra from the regions 0.79 to 4.62 ppm, 4.60 to 5.43 ppm, and 5.09 to 8.49 ppm, respectively of different palm date flesh as (a, e, p) for DNF, (b, f, q) for SRF, (c, g, r) for ARF, and (d, h, s) for AJF recorded by using solution-state NMR of 800 MHz in D2O solution.

**
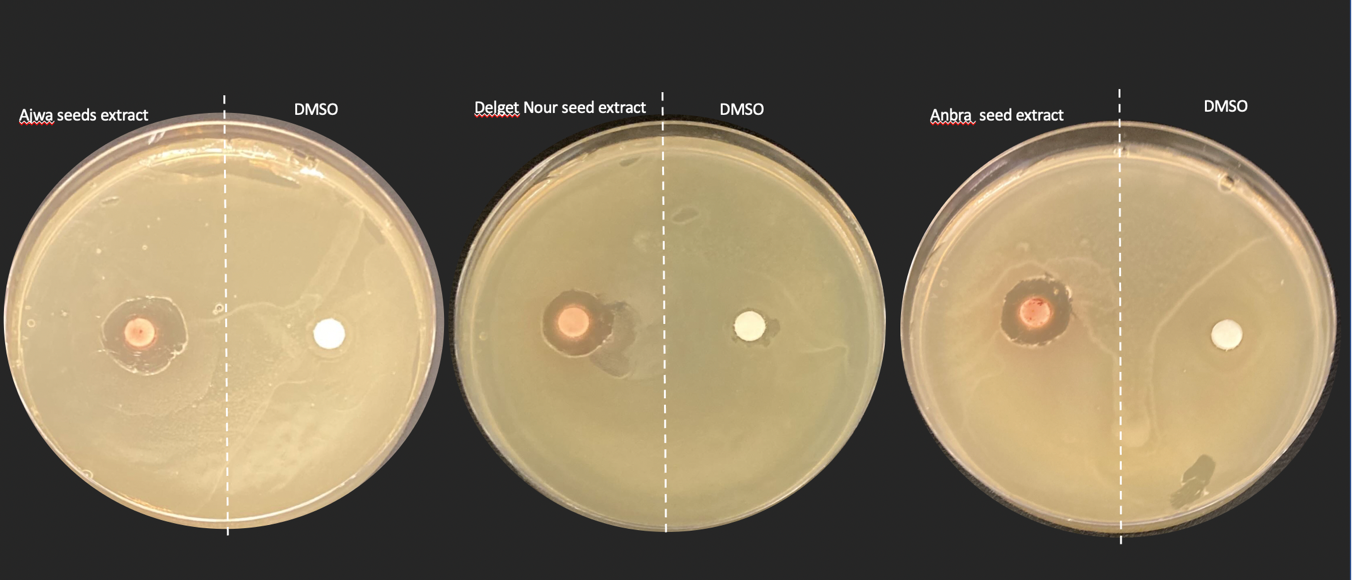
**

**Figure S8.** Antibacterial activity of different seed extracts with gram-negative bacteria (E. coli). DMSO was used as a negative control. Examples of inhibition zones in MH- agar plates for different date seed methanol extracts.
